# Frontotemporal dementia causative CHMP2B impairs neuronal endolysosomal traffic-rescue by *TMEM106B* knockdown

**DOI:** 10.1093/brain/awy284

**Published:** 2018-11-29

**Authors:** Emma L Clayton, Carmelo Milioto, Bhavana Muralidharan, Frances E Norona, James R Edgar, Armand Soriano, Paymaan Jafar-nejad, Frank Rigo, John Collinge, Adrian M Isaacs

**Affiliations:** 1 Department of Neurodegenerative Disease, UCL Institute of Neurology, Queen Square, London, UK; 2 Department of Clinical and Experimental Epilepsy, UCL Institute of Neurology, Queen Square, London, UK; 3 UK Dementia Research Institute at UCL, UCL Institute of Neurology, Queen Square, London, UK; 4 Cambridge Institute for Medical Research, University of Cambridge, Cambridge, UK; 5 Ionis Pharmaceuticals, Carlsbad, California, USA; 6 MRC Prion Unit at UCL, Institute of Prion Diseases, Queen Square, London, UK

**Keywords:** frontotemporal dementia, trafficking, ESCRT, CHMP2B, TMEM106B

## Abstract

Mutations in the endosome-associated protein CHMP2B cause frontotemporal dementia and lead to lysosomal storage pathology in neurons. We here report that physiological levels of mutant CHMP2B causes reduced numbers and significantly impaired trafficking of endolysosomes within neuronal dendrites, accompanied by increased dendritic branching. Mechanistically, this is due to the stable incorporation of mutant CHMP2B onto neuronal endolysosomes, which we show renders them unable to traffic within dendrites. This defect is due to the inability of mutant CHMP2B to recruit the ATPase VPS4, which is required for release of CHMP2B from endosomal membranes. Strikingly, both impaired trafficking and the increased dendritic branching were rescued by treatment with antisense oligonucleotides targeting the well validated frontotemporal dementia risk factor *TMEM106B*, which encodes an endolysosomal protein. This indicates that reducing TMEM106B levels can restore endosomal health in frontotemporal dementia. As *TMEM106B* is a risk factor for frontotemporal dementia caused by both *C9orf72* and progranulin mutations, and antisense oligonucleotides are showing promise as therapeutics for neurodegenerative diseases, our data suggests a potential new strategy for treating the wide range of frontotemporal dementias associated with endolysosomal dysfunction.

## Teaser

Mutations in the endosome-associated protein CHMP2B cause frontotemporal dementia (FTD). Clayton *et al.* report a mechanism for CHMP2B-mediated neuronal dysfunction and a potential therapeutic strategy targeting the *TMEM106B* gene, a major risk factor for FTD. Targeting *TMEM106B* may be relevant to a broad range of FTDs.

**See Bechek and Gitler (doi:10.1093/brain/awy294) for a scientific commentary on this article.**

## Introduction

A mutation in *CHMP2B*, a subunit of the endosomal sorting complex required for transport-III (ESCRT-III), causes an autosomal dominant form of frontotemporal dementia (FTD) in a Danish cohort (Skibinski *et al.*, 2005; Lindquist *et al.*, 2008). The multi-subunit complexes ESCRTs 0-III are involved in membrane budding events essential for diverse cellular functions including the final stages of cell division (Carlton and Martin-Serrano, 2007), egress of viruses from cells (Morita and Sundquist, 2004), nuclear envelope reformation after mitosis (Olmos *et al.*, 2015; Vietri *et al.*, 2015), and importantly for this study, the formation and fission of intra-luminal vesicles in late endosomes/multivesicular bodies (reviewed in Raiborg and Stenmark, 2009; Schuh and Audhya, 2014). Proteins within intra-luminal vesicles are then delivered to lysosomes for degradation via endo-lysosomal fusion.

FTD is a common form of young-onset dementia (Ratnavalli *et al.*, 2002; Harvey *et al.*, 2003) characterized by atrophy of the frontal and temporal lobes. FTD presents with personality, behaviour and language changes (Neary *et al.*, 1998; McKhann *et al.*, 2001). The FTD causative mutation in *CHMP2B* occurs in a splice acceptor site, which results in the production of two C-terminally truncated variants of the protein. The final 36 amino acids are either replaced by a single valine residue (termed CHMP2B^Intron5^), or by 29 nonsense residues (CHMP2B^Δ10^) (Skibinski *et al.*, 2005), with CHMP2B^Intron5^ responsible for driving neurodegeneration (Lee *et al.*, 2007; Ghazi-Noori *et al.*, 2012; Gascon *et al.*, 2014). In addition to *CHMP2B*, several genetic mutations are known to cause FTD. Mutations in the genes that encode tau (*MAPT*), progranulin (*GRN*) and *C9orf72* are the most common causes of FTD, while additional rare mutations have been identified in valosin-containing protein (*VCP*), TDP-43 (*TARDBP*), fused in sarcoma (*FUS*) (Rohrer and Warren, 2011) and TANK- binding kinase 1 (*TBK1*) (Gijselinck *et al.*, 2015; Le Ber *et al.*, 2015; Pottier *et al.*, 2015; van der Zee *et al.*, 2017). Interestingly, FTD shares both common genetic causes and pathologies with another neurodegenerative condition, amyotrophic lateral sclerosis (ALS) (Bennion and Pickering-Brown, 2014).

A key question in the aetiology of FTD is how genes with such diverse functions result in the specific degeneration of cortical neurons. Recent work has begun to suggest that these FTD causative genes converge on dysfunction of the endolysosomal system. Heterozygous mutations in *GRN*, a lysosomal localized protein, cause FTD and lead to biochemical changes characteristic of lysosomal storage diseases (Gotzl *et al.*, 2014); with rare homozygous *GRN* mutations directly causing neuronal ceroid lipofuscinosis, a lysosomal storage disorder (Smith *et al.*, 2012). We recently showed that *CHMP2B* mutation also leads to lysosomal storage pathology (Clayton *et al.*, 2015). Further to this, TMEM106B, a risk factor for FTD (Van Deerlin *et al.*, 2010; van der Zee and Van Broeckhoven, 2011), is located at the endolysosome and directly affects endolysosomal function, including dendritic endolysosomal trafficking (Brady *et al.*, 2013; Schwenk *et al.*, 2014; Stagi *et al.*, 2014; Klein *et al.*, 2017). We now report that mutation in *CHMP2B* causes a decrease in neuronal endolysosomal motility, which is accompanied by increased dendritic branching. We show that the physical incorporation of mutant CHMP2B into an endolysosomal vesicle renders that organelle stationary. Strikingly, we found that both the trafficking and dendritic branching defects could be reversed by knockdown of the FTD risk factor *TMEM106B*, suggesting that reduction of TMEM106B levels may be a broadly applicable therapeutic target for FTD.

## Materials and methods

### Mice

Mice were housed in a category 3 SPF facility in individually ventilated cages under negative pressure in groups of three to five animals with environmental condition targets of: temperature 20 ± 2°C, relative humidity 55% ± 10%, 12:12-h photoperiod. Mice were provided with water and pelleted diet *ad libitum*. All cages are provided with environmental enrichment in the form of nesting material, chew blocks and mouse houses. Procedures were carried out under UK Home Office Project Licence 7009014.

The previously described mutant CHMP2B^Intron5^ expressing mouse line Tg153 (Ghazi-Noori *et al.*, 2012) was backcrossed over 10 generations to C57Bl6J, and was maintained as a homozygous line. GFP-LC3 mice (Mizushima *et al.*, 2004) were obtained from Riken BRC. Homozygous GFP-LC3 mice were crossed to homozygous mutant CHMP2B^Intron5^ mice to initially generate mice heterozygous for both transgenes. Mice heterozygous for both genes were then crossed to produce animals homozygous for both GFP-LC3 and CHMP2B^Intron5^, generating the double homozygous GFP-LC3 X CHMP2B^Intron5^ line. Homozygosity for both transgenes was verified by genotyping. Double homozygotes occurred in approximately Mendelian ratios.

### Immunoblotting

The olfactory bulb and cerebellum were removed from whole brains of postnatal Day 0 or Day 1 mice. Homogenates were prepared in Dulbecco’s phosphate-buffered saline (D-PBS) containing cOmplete EDTA-free protease inhibitors (Roche) using a TissueRuptor® (Qiagen) to make a 10% w/v solution. Following a 2-min 200*g* spin to pellet debris, the supernatant was resuspended in D-PBS. Benzonase® (Novagen) was added to digest DNA and the homogenates were incubated at 4°C for 1 h. Laemmli sample buffer (2×) was added and the samples were heated at 100°C for 10 min prior to sodium dodecyl sulphate polyacrylamide gel electrophoresis. Samples were run on 10% Bis-Tris gels (Life Technologies) with MES buffer, then transferred onto polyvinylidene fluoride, blocked with 5% bovine serum albumin in PBS-T, and probed using anti-CHMP2B (Ghazi-Noori *et al.*, 2012). Loading controls were performed using mouse anti-β-actin (A5441, Sigma). Detection was performed with horseradish peroxidase-conjugated secondary antibodies and SuperSignal™ West Pico Chemiluminescent Substrate (Thermo Scientific). Quantification was performed using ImageJ software. Band intensity was normalized to the indicated loading control, and averages taken of three mice per genotype.

### Pre-embedding labelling of mouse brains for electron microscopy

Mice aged to 6 months were perfusion fixed with 4% paraformaldehyde (PFA) and the brains removed and post-fixed overnight in the same fixative. One hundred-micrometre thick sections were cut using a vibratome. Areas of interest were dissected and refixed with 2% PFA/2.5% glutaraldehyde in 0.1 M cacodylate buffer. Thick sections were washed with HEPES-buffered saline (HBS) pH 7.4 before being permeabilized in HBS containing 0.05% Triton™ X-100 and 10% bovine serum albumin (BSA). Tissue was incubated with primary anti-GFP antibody (ab6556, Abcam) overnight at 4°C in HBS with 0.005% Triton™ X-100 and 1% BSA. Tissue was then washed before being incubated with anti-rabbit nanogold secondaries (Nanoprobes). The tissue was washed before being refixed with 2% PFA/2.5% glutaraldehyde in 0.1 M cacodylate buffer. Nanogold particles were then enhanced using GoldEnhance (Nanoprobes). The tissue was then post-fixed with 1% osmium tetroxide before being dehydrated with ethanol, and embedded in Araldite epoxy resin (Agar Scientific). Ultrathin sections (70 nm) were cut using a diamond knife mounted to a Reichert Ultracut S ultramicrotome (Leica) and picked up onto coated electron microscopy grids. The sections were stained with lead citrate and observed in a FEI Tecnai Spirit transmission electron microscope at an operating voltage of 80 kV. In conventional electron microscopy, neurons were identified by their characteristic large nuclei with a clear nucleolus and electron lucent cytoplasm.

### Cortical culture and transfection

Primary cortical cultures were prepared from mice of either sex (postnatal Days 0 or 1). Briefly, the cortices were dissected, digested in trypsin (Sigma) and triturated with a fine fire polished Pasteur pipette to achieve a single cell suspension. Cells were plated in a minimal volume of Dulbecco’s modified Eagle’s medium supplemented with 10% foetal bovine serum, 1% penicillin/streptomycin and 1% GlutaMAX™ (all Invitrogen), at a density of 1000 cells/mm^2^ on coverslips or live cell imaging dishes coated with poly-d-lysine (Sigma). One to two hours after plating, maintenance medium of Neurobasal™-A containing 2% B27, 0.25% penicillin/streptomycin and 0.25% GlutaMAX™ (all Invitrogen) was added to the cells. Neurons were cultured at 37°C and 5% CO_2._ For transfection, cultures were incubated with a mix of DNA and Lipofectamine® 2000 in serum-free Neurobasal™-A for 30 min. Cultures were washed twice with serum-free Neurobasal™-A, then returned to conditioned maintenance medium. Cultures were either fixed for immunostaining or used for live cell imaging 12–16 h after transfection.

### Immunofluorescence

Neurons were fixed in 4% PFA/PBS for 10 min, then permeabilized in 0.5% Triton™ X-100/PBS for 5 min. For blocking, 3% bovine serum albumin/PBS was used for 30 min. Primary antibodies were diluted in PBS and incubated at room temperature for 1 h. Coverslips were washed in PBS three times for 5 min before the addition of Alexa Fluor® conjugated secondary antibodies (Life Technologies) for 1 h. Coverslips were then washed three times for 5 min in PBS, and mounted in ProLong™ Gold Antifade Mountant with DAPI (Life Technologies). All steps were carried out at room temperature. Images were collected using a 40× oil lens with 1.4 NA on a Zeiss LSM 710. Primary antibodies used were LAMP2 (Abl-93, University of Iowa Hybridoma Bank), β-tubulin (5568, Cell Signalling), HA (3F10, Sigma) and MAP2 (ab5392, Abcam).

### Live cell imaging

All recordings were made at 1 frame/s. Live cell imaging was conducted in HEPES-buffered phenol free maintenance medium, to reduce background fluorescence. For LysoTracker® imaging, cortical cultures in live cell imaging dishes were incubated with 100 nm LysoTracker® Red DND-99 (Thermo Fisher) in conditioned medium at 37°C for 20 min then washed twice with fresh imaging medium before imaging. GFP-LAMP transfected cells were transferred to imaging medium for live cell recordings. Live cell recordings were made on a heated stage. Kymographs were generated and analysed using the Multiple Kymograph plugin for ImageJ. Dendrites were distinguished from axons by their distinctive morphology.

For FRAP recordings (fluorescence recovery after photobleaching), images were recorded for 10 s prior to bleaching to establish baseline fluorescence. A single circular region of interest within the cell was bleached with a laser pulse, and then recording continued for 120 frames post-bleach. FRAP plots were generated in ImageJ by quantifying the fluorescence intensity in the bleached region of interest over time normalized to the starting fluorescent intensity. Recordings were made on LSM 710, except Fig. 8C and Supplementary Fig. 8, which were recorded on LSM 880.

### Neurite outgrowth

Maximum intensity projections of zsGreen transfected cells were generated by capturing *z*-stacks through the entire neuritic projection of primary cortical cultures fixed at 7 days *in vitro* (DIV) 24 h after transfection. Neuritic arbours were traced in NeuronJ, and Sholl analysis performed on neuronal tracings in ImageJ using 10 µm stepped radii from the cell soma. Images were captured on LSM 710, or LSM 880 for Supplementary Fig. 8.

### *Tmem106b* antisense oligonucleotides

Antisense oligonucleotides (ASOs) against *Tmem106b* and control ASOs were provided by Ionis Pharmaceuticals. ASOs were synthesized as previously described (Swayze *et al.*, 2007) and were 20 bp in length, with five 2′-*O*-methoxyethyl-modified nucleotides at each end of the oligonucleotide, 10 DNA nucleotides in the centre. The backbone of the ASOs consists of a mixture of phosphorothioate (PS) and phosphodiester (PO) linkages: 1-PS, 4-PO, 10-PS, 2-PO and 2-PS (5′ to 3′). CNTL ASO 676630, 5′-CCTATAGGACTATCCAGGAA-3′; ASO 687524, 5′-GTTCTCCATGAATAATAGGC-3′; ASO-687552, 5′-GCACTTTATTTACAATATTG-3′.

### Quantification of knockdown

Mixed primary cortical neuronal cultures were prepared from embryonic Day 14 C57Bl/6 mice and plated in 6-well plates coated with poly-d-lysine (1.5 million cells per well). Twenty-four hours after plating the cells ASOs were added to the media. Cells were collected 48 h after ASO treatment.

For quantification at the RNA level cells were homogenized in 200 µl RLT using the Qiagen RNeasy® Kit (Qiagen) containing 1% 2-mercaptoethanol. Total RNA was purified further using a mini-RNA purification kit (Qiagen). After quantitation, the RNA samples were subjected to real time RT-PCR analysis. The Life Technologies ABI StepOne Plus™ Sequence Detection System (Applied Biosystems) was used. Briefly, 30-µl RT-PCR reactions containing 10 µl of RNA were run with the RNeasy® 96 kit reagents and the primer probe sets listed in the materials section. All real time RT-PCR reactions were run in triplicate. The expression level of *Tmem106b* mRNA was normalized to that of *Gapdh* mRNA, and this was further normalized to the level measured in controls that were treated with control ASOs. Expression data are reported as per cent of control.

For quantification of knockdown at the protein level, cells were lysed in RIPA buffer (Thermo Fisher, 89900) supplemented with protease inhibitor (cOmplete™ Lysis-M EDTA-free Roche, 45-4719964001). NuPAGE™ LDS Sample Buffer (4×) (Invitrogen, NP0007) and 1% 2-mercaptoethanol was added to the whole cell lysate without spinning down. The samples were left at room temperature for 30 min and run on NuPAGE™ MOP 12-well gels. Anti-Tmem106b (Bethyl Laboratories, A303-439A) was used at 1:1000. Secondary antibody was goat anti-rabbit HRP (Cell Signaling, 7074S).

### Statistical analysis

Statistical analysis was performed with Graphpad Prism software. Statistical tests used are indicated in the figure legends.

### Data availability

The data that support the findings of this study are available from the corresponding author, upon reasonable request.

## Results

### Reduction of lysosomes in the soma of primary cortical neurons expressing CHMP2B^Intron5^ at physiological levels

**Figure 1:**
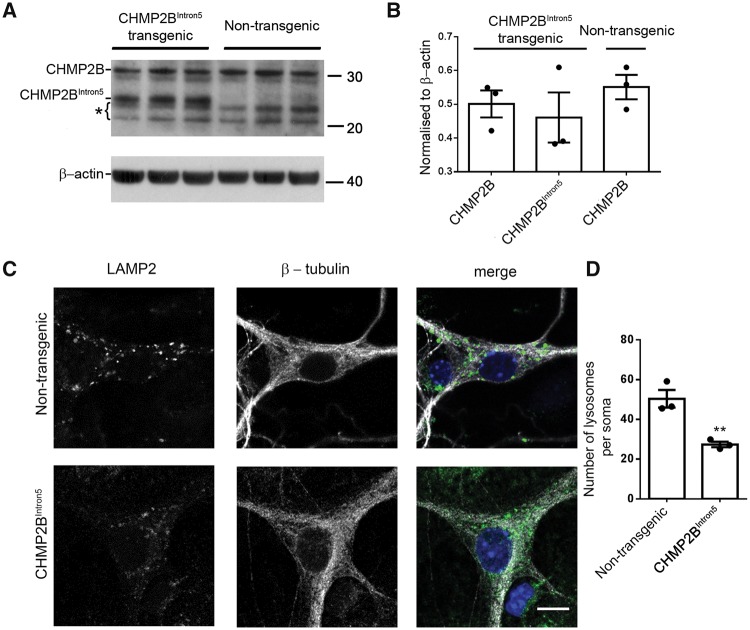
**Physiological expression of mutant CHMP2B results in a decrease of endolysosomes at the soma of cortical neurons.** (**A**) Western blot of CHMP2B levels on brain homogenates from P0 or P1 postnatal mutant CHMP2B mice or non-transgenic controls. β-actin is shown as a loading control. Bracket indicates non-specific bands. (**B**) Quantification of bands shown in **A** relative to loading controls. (**C**) LAMP2 staining in mutant CHMP2B and control β-tubulin stained cortical neurons. Scale bar = 10 µm. (**D**) Quantification of LAMP2-positive structures in DIV 14–16 cortical neurons. *n =* 3, with 6–10 neurons per *n*. Unpaired *t*-test, **P < 0.01.

To determine the early changes driving the neuronal lysosomal storage pathology we previously observed in adult CHMP2B^Intron5^ mouse brain (Clayton *et al.* 2015), we investigated the endolysosomal system in postnatal primary cortical neurons derived from CHMP2B^Intron5^ mice. Mature/late endosomes and lysosomes share characteristics, including the same membrane markers and acidic pH, thus we use the term endolysosome here to encompass both late endosomes and lysosomes. First, we confirmed that mutant CHMP2B is expressed at physiological levels in our primary culture system, as it is in adult brain in this model (Ghazi-Noori *et al.* 2012). Indeed, quantification of CHMP2B expression in postnatal cortical homogenates shows that mutant CHMP2B is expressed at a level equal to endogenous mouse CHMP2B (Fig. 1A and B). Initially we investigated the effect of mutant CHMP2B on the number of lysosomes in fixed primary neuronal mouse cortical cultures. Immunostaining for LAMP2 revealed a significant reduction in lysosome number in the soma of mutant CHMP2B primary neurons at DIV 14–16 (Fig. 1C and D), and also at DIV 21 (Supplementary Fig. 1). No significant change was seen in the size of LAMP2 positive structures (Supplementary Fig. 1C and D).

### Autophagosomes are not altered in CHMP2B^Intron5^ primary neurons or adult brain

Autophagosome accumulation has previously been reported in several different mutant CHMP2B over-expression cell models (Filimonenko *et al.*, 2007; Lee *et al.*, 2007; Lee and Gao, 2009). Therefore, we investigated whether in addition to a reduction in endolysosomes, an early defect in autophagy is caused by physiological levels of mutant CHMP2B in primary neurons. Under basal or starvation conditions, no significant difference was seen in the number or size of LC3- (Supplementary Fig. 2A–D) or WIPI2-positive (Supplementary Fig. 3) autophagosomes in CHMP2B^Intron5^ cultures compared to control cultures. To investigate autophagosomes *in vivo*, we crossed our CHMP2B mutant mice with GFP-LC3 mice. At 6 months of age, immuno-electron microscopy for GFP was able to successfully label double-membrane structures, indicative of autophagosomes in mutant CHMP2B × GFP-LC3 mouse brains (Supplementary Fig. 2E), confirming the staining protocol was effective. However, we did not observe any labelling of GFP-LC3 on the mutant CHMP2B-induced lysosomal storage deposits we previously reported (Clayton *et al.*, 2015) (Supplementary Fig. 2F). These data indicate that endolysosomal, rather than autophagy, defects are the earliest pathology we can detect in our mutant CHMP2B mouse model.

### Endolysosomal trafficking is impaired in CHMP2B^Intron5^ primary cortical neurons

**Figure 2:**
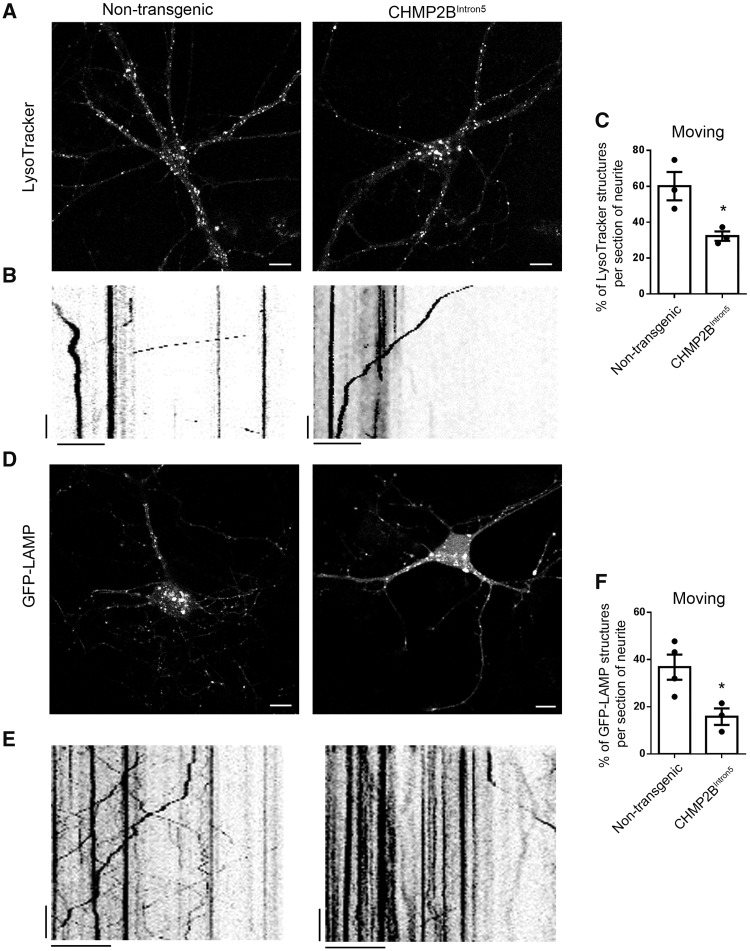
**Mutant CHMP2B decreases endolysosomal trafficking.** (**A**) Representative images of LysoTracker® labelled control and mutant CHMP2B cultures. Scale bar = 10 µm. (**B**) Example kymographs from 50 µm sections of dendrite labelled with LysoTracker® and imaged for 120 s. Vertical scale bar = 20 s, horizontal scale bar = 10 µm. (**C**) Quantification of moving and stationary LysoTracker® structures from kymographs. *n =* 3, with three to five neurons per *n*, DIV 12–20 cortical cultures. (**D**) Representative images of GFP-LAMP transfected control and mutant CHMP2B cortical cultures. Scale bar = 10 µm. (**E**) Kymographs from sections of dendrite labelled with GFP-LAMP and live cell imaged for 120 s. Vertical scale bar = 20 s, horizontal scale bar = 10 µm. (**F**) Quantification of moving and stationary GFP-LAMP structures from kymographs. *n =* 3 for mutant CHMP2B, *n =* 4 for non-transgenic with four to seven neurons per *n*, DIV 10–14 cortical cultures. Unpaired *t*-test, *P < 0.05.

Having verified that endolysosomal defects are the earliest pathological event we detect in mutant CHMP2B neurons, we investigated the nature of this defect further. We used live-imaging to investigate the trafficking dynamics of endolysosomes. Initially, we analysed LysoTracker®, a dye that specifically labels acidic organelles, in the dendrites of mutant and control non-transgenic neurons (Fig. 2A). Kymographs were generated to represent the movement of LysoTracker®-labelled vesicles (Fig. 2B), and the proportion of stationary versus moving vesicles quantified in mutant CHMP2B and control neurons. Mutant CHMP2B dendrites contained a significantly smaller population of moving vesicles than control dendrites [30.9 ± 2.7% versus 59.3 ± 7.9% (mean ± standard error of the mean, SEM)] (Fig. 2C). This was confirmed with a second endolysosomal marker, GFP-LAMP1 (Fig. 2D–F). Mutant CHMP2B dendrites contained a significantly smaller proportion of moving LAMP1 positive vesicles than control dendrites [15.8 ± 3.5% versus 36.8 ± 5.3% (mean ± SEM)] (Fig. 2F). Live cell imaging of both LysoTracker® and GFP-LAMP1 revealed that mutant CHMP2B neurons have defective trafficking of endolysosomes, with a significantly greater proportion of stationary endolysosomes. The proportion of moving vesicles that trafficked in retrograde or anterograde directions was not significantly different to control (Supplementary Fig. 4), indicating a general decrease in endolysosomal transport that was not specific for direction of travel.

### Mutant CHMP2B^Intron5^ is unable to dissociate from stationary endolysosomes

We hypothesized that mutant CHMP2B is unable to dissociate from the endolysosomal membrane, thus exacerbating its detrimental effects and leading to the impairment in trafficking that we observed. To test this possibility we used the GFP-containing localization and affinity (LAP) tag to label mutant CHMP2B. The LAP tag incorporates a long, flexible linker sequence between GFP and the tagged protein and has been shown not to affect the function of several CHMP proteins, including CHMP2B (Mierzwa *et al.*, 2017).

**Figure 3:**
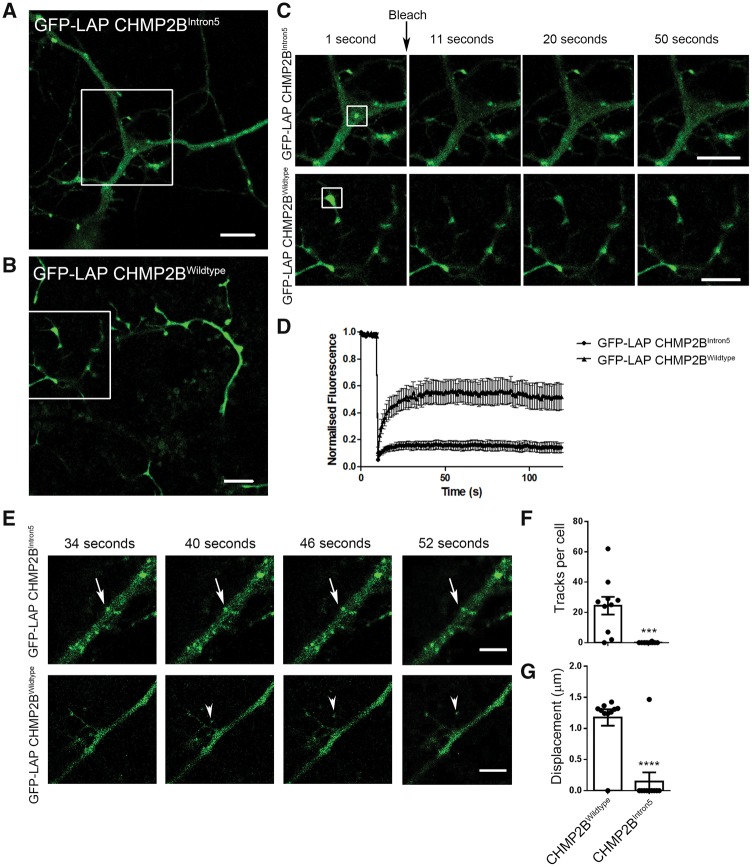
**Mutant CHMP2B structures are stationary and show no fluorescence recovery after photobleaching.** GFP-LAP tagged mutant (**A**) or wild-type (**B**) CHMP2B in DIV 10 primary cortical cultures. (**C**) *Insets* from **A** and **B**. The structures highlighted by boxes were bleached at the indicated time point, and the recovery of fluorescence followed over time. Representative time points are shown (1, 11, 20 and 50 s). (**D**) Quantification of the average fluorescence recovery over time for mutant CHMP2B (circles) or wild-type CHMP2B (triangles) normalized to the starting fluorescence. (**E**) Representative time points of immobile GFP-LAP CHMP2B^Intron5^ structures (arrows) and moving GFP-LAP CHMP2B^Wildtype^ structures (arrowheads). (**F**) Quantification of the number of traces per transfected cell generated using Trackmate in ImageJ. (**G**) Quantification of the average displacement of traces automatically generated in ImageJ. *n =* 10 DIV 10 neurons from two independent experiments. Unpaired *t*-test, ***P < 0.001, ****P < 0.0001. Scale bars = 5 µm.

Cortical neurons transfected with mutant GFP-LAP CHMP2B showed a punctate localization of the mutant protein (Fig. 3A). This distribution of fluorescence is in contrast to the neurons labelled with wild-type GFP-LAP CHMP2B, which typically show a cytosolic distribution (Fig. 3B). This distribution is similar to that described previously for wild-type and mutant CHMP2B (Belly *et al.*, 2010; Urwin *et al.*, 2010), which indicates that the GFP-LAP tag does not affect the distribution of CHMP2B.

To assess the motility of mutant and wild-type CHMP2B, we performed fluorescence recovery after photobleaching (FRAP) on discrete structures in the dendrites of transfected neurons (Fig. 3C). Strikingly, mutant CHMP2B structures show no recovery of fluorescence after photobleaching (Fig. 3C, top). This is in sharp contrast to the recovery after bleaching of wild-type CHMP2B (Fig. 3C, bottom), which shows a rapid recovery of fluorescent signal (time to 50% recovery of wild-type fluorescence = 25 s) (Fig. 3D). These data indicate that while wild-type CHMP2B is freely dissociating within the environment of the dendrite, mutant CHMP2B is statically localized to stationary structures with no turnover of the mutant ESCRT subunit.

### Incorporation of CHMP2B^Intron5^ onto endolysosomes renders them unable to traffic

To determine whether this stable incorporation of mutant CHMP2B onto endosomes renders them unable to traffic, we measured the movement of mutant and wild-type GFP-LAP CHMP2B vesicles in real time. CHMP2B^Intron5^ labelled structures are stationary over time (Fig. 3E, arrows). However, small CHMP2B^Wildtype^ structures were occasionally observed to appear and traffic for a short distance (Fig 3E, arrowheads). Automated tracking of these events detected numerous displacement events of CHMP2B^Wildtype^ labelled puncta per neuron, with only one trafficking event observed in the CHMP2B^Intron5^ labelled neurons (Fig 3F and G). These data directly show that mutant CHMP2B positive endolysosomes are unable to traffic.

### CHMP2B^Intron5^ vesicles do not recruit the ESCRT dissociation factor VPS4A

**Figure 4:**
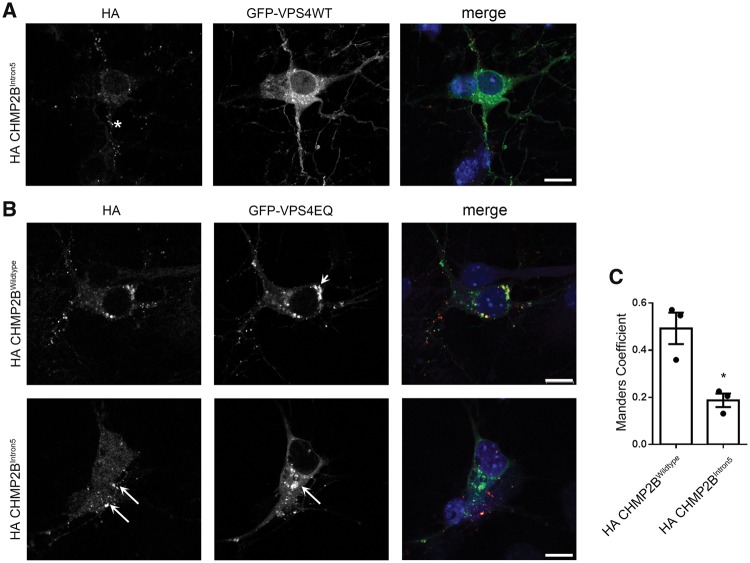
**Mutant CHMP2B does not recruit VPS4.** (**A**) Representative examples of neurons co-transfected with haemagglutinin (HA) tagged CHMP2B and GFP-VPS4. Asterisk indicates an example of localization of mutant HA-CHMP2B, which is negative for VPS4. (**B**) Representative examples of neurons co-transfected with GFP-VPS4EQ and HA-tagged wild-type (*top*) or mutant CHMP2B (*bottom*). Arrowhead (*top*) indicates area of co-localization of wild-type CHMP2B with VPS4EQ. Arrows (*bottom*) show differential localization of mutant CHMP2B and VPS4EQ. DIV 8 cortical cultures. (**C**) Manders coefficient of co-localization generated using JaCoP in ImageJ. Unpaired *t*-test, *P < 0.05. Scale bars = 10 µm.

Next, we investigated why mutant CHMP2B is statically localized to stationary endolysosomes. The key step in dissociation of ESCRT components from the endolysosomal membrane after intra-luminal vesicle formation is the recruitment of the ATPase VPS4 by CHMP2A and/or CHMP2B (Stuchell-Brereton *et al.*, 2007; Lata *et al.*, 2008; Wollert *et al.*, 2009). Therefore, we investigated whether recruitment of VPS4 is compromised by the presence of mutant CHMP2B, which lacks the microtubule-interacting and transport (MIT) interacting motif (MIM) necessary for recruitment of VPS4 (Stuchell-Brereton *et al.* 2007). We co-transfected primary cortical neurons with mutant CHMP2B and GFP-VPS4 variants and as expected, mutant CHMP2B structures do not recruit VPS4 (Fig. 4A). To verify this finding further, we used the ATPase deficient variant of VPS4, VPS4EQ. This mutant is unable to dissociate from the formed intra-luminal vesicle, which leads to the formation of enlarged endosomes (Bishop and Woodman, 2000). Wild-type CHMP2B co-localized with these GFP-VPS4EQ positive structures (Fig. 4B, short arrow, top), while significantly less mutant CHMP2B co-localized with GFP-VPS4EQ (Fig. 4B, long arrows, bottom). These data show that mutant CHMP2B is unable to recruit VPS4, which explains its inability to dissociate from endolysosomes within neurons.

### Mutant CHMP2B^Intron5^ causes increased dendritic branching

**Figure 5:**
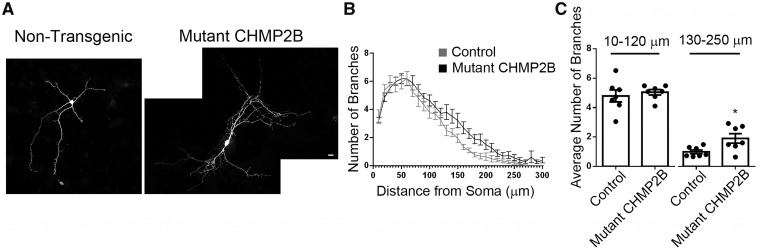
**Peripheral neurite branching is increased in mutant CHMP2B neurons.** (**A**) Representative maximum intensity projections of DIV 7 non-transgenic control and mutant CHMP2B neurons as labelled. (**B**) Sholl analysis of neurite branching for control and mutant CHMP2B neurons. (**C**) Average number of branches per Sholl intersection for 10–120, and 130–250 µm from the cell soma. Control data are shown in grey, mutant CHMP2B in black. Unpaired *t*-test, *P < 0.05. *n =* 7 for both, with four to six neurons per *n*. Scale bar = 10 µm.

To determine whether there was a functional consequence of altered endolysosomal trafficking in mutant CHMP2B dendrites, we investigated dendritic branching. Sholl analysis revealed that physiological levels of mutant CHMP2B causes an increase in distal dendritic branching in primary neurons (Fig. 5). Remarkably, these data show that mutant CHMP2B has the opposite effect in dendrites to those caused by reduced levels of the FTD risk factor TMEM106B, which has been found to increase the transport of dendritic lysosomes and to also decrease dendritic branching (Schwenk *et al.*, 2014; Stagi *et al.*, 2014).

### *Tmem106b* knockdown restores endolysosomal trafficking and branching defects in mutant CHMP2B^Intron5^ neurons

**Figure 6:**
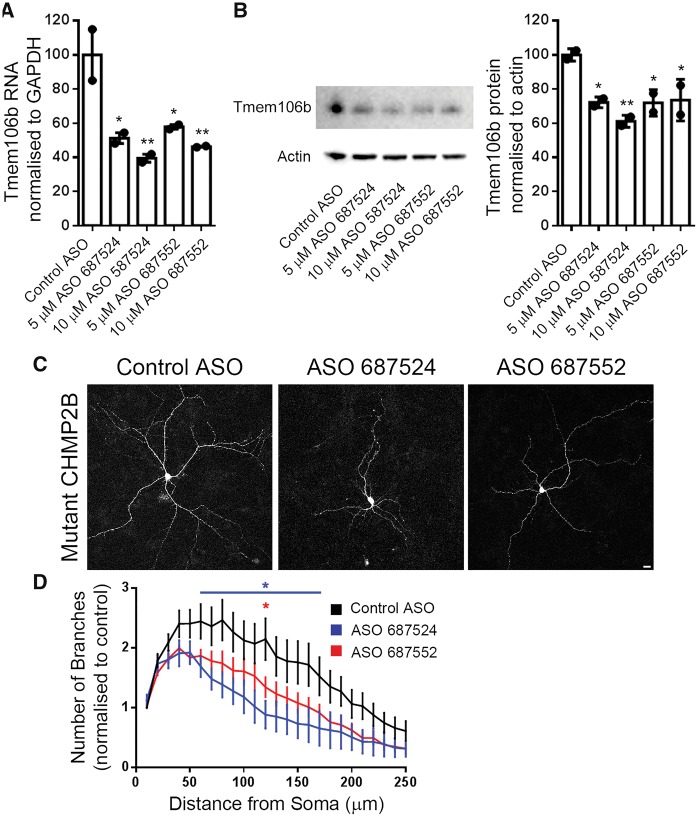
**Knockdown of *Tmem106b* with ASOs rescues neuritic branching and endolysosomal trafficking defects.** (**A**) Quantification of *Tmem106b* mRNA levels in mutant CHMP2B cortical cultures following treatment with the indicated ASOs normalized to GAPDH, *n =* 2. (**B**) Blot and quantification of TMEM106B protein levels from primary cultures treated with the indicated ASOs. (**C**) Representative maximum intensity projections of mutant CHMP2B neurons at DIV 7 treated with 5 µM of the indicated ASOs for 7 days. Scale bar = 10 µm. (**D**) Sholl analysis of ASO treated neurons normalized to control ASO treated neurons. ASO 687524 data are shown in blue, ASO 687552 in red. Two-way ANOVA with Dunnett’s multiple comparisons. *P < 0.05. Blue asterisks indicate statistical significance between control ASO and ASO 687524. Red asterisks indicate significance between control ASO and ASO 687552. *n =* 6 for each condition, with 4–10 neurons per *n*.

**Figure 7:**
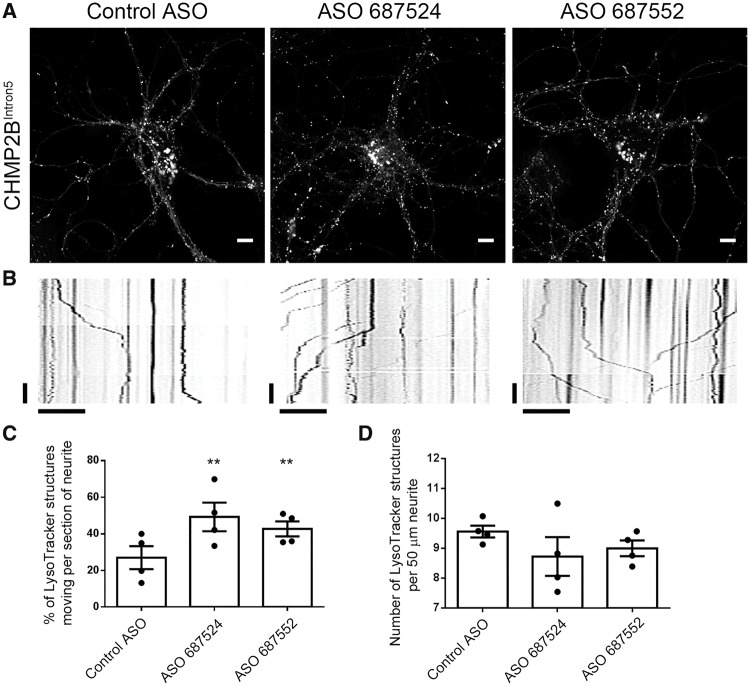
**Knockdown of *Tmem106b* with ASOs rescues endolysosomal trafficking defects.** (**A**) Representative images of mutant CHMP2B neurons treated with 5 µM of the indicated ASOs for 7 days loaded with LysoTracker®. Scale bar = 10 µm. (**B**) Representative kymographs from LysoTracker® live cell recordings of neurons labelled with the indicated ASOs. Vertical scale bar = 20 s, horizontal scale bar = 10 µm. (**C**) Quantification of kymographs of LysoTracker® movement in DIV 14 mutant CHMP2B cortical cultures following treatment with the indicated ASOs. *n =* 4 with five to seven neurons per *n*. (**D**) Quantification of the number of LysoTracker® labelled structures in the sections of neurite chosen for kymograph analysis. *n =* 4 with five to seven neurons per *n*. One-way ANOVA with Newman-Keuls multiple comparison test, **P < 0.01.

Therefore, we reasoned that knockdown of *Tmem106b* might be sufficient to rescue the dendritic branching and lysosomal trafficking defects in mutant CHMP2B neurons. Quantification of a fluorescently labelled non-targeting control ASO showed that 3 days after ASO treatment, 90% of neurons contain fluorescent ASOs, and 80% at 7 days post-treatment (Supplementary Fig. 5), indicating efficient entry and retention of ASOs in primary cortical cultures. Two distinct murine ASOs were used to knockdown *Tmem106b* in mutant CHMP2B neurons by ~50% at the RNA level and up to 40% at the protein level after 2 days (Fig. 6A and B), and knockdown was maintained at 7 days (65–70% knockdown at the RNA level, Supplementary Fig. 6). Knockdown of *Tmem106b* with either ASO rescued the increase in dendritic branching seen in mutant CHMP2B neurons (Fig. 6C and D). Strikingly, knockdown of *Tmem106b* also rescued the LysoTracker® trafficking defect (Fig. 7A and B), with two independent ASOs causing a significant increase in the proportion of moving LysoTracker® labelled structures in mutant CHMP2B neurons (Fig. 7C). This effect was specifically due to increased trafficking as the number of LysoTracker® structures within dendrites was not affected (Fig. 7D). Interestingly, *Tmem106b* ASO treatment of control neurons had more modest effects, with a trend towards increased trafficking of LysoTracker® structures but no effect on dendrite branching (Supplementary Fig. 7).

**Figure 8:**
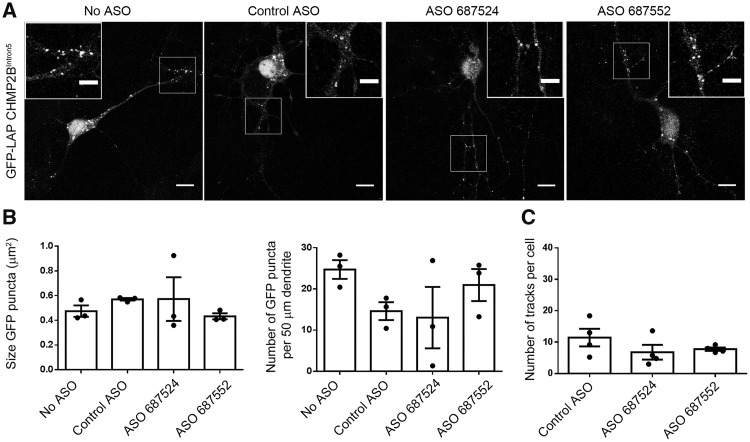
**The size, number and motility of GFP-LAP CHMP2B^Intron5^ puncta is not altered in TMEM106B ASO-treated neurons.** (**A**) Representative images of neurons transfected with GFP-LAP CHMP2B^Intron5^ and treated with 5 µM of the indicated ASOs. Scale bar = 10 µm. (**B**) Quantification of the size of GFP puncta, and quantification of the number of GFP puncta per 50 µm section of proximal dendrite in fixed neurons. *n =* 3 with 3–10 DIV 10 neurons per *n*. (**C**) Quantification of the number of motile structures in live-imaged GFP-LAP CHMP2B^Intron5^ transfected cells treated with the indicated ASOs. *n =* 3 with five DIV 10–11 neurons per *n*.

To investigate whether *Tmem106b* knockdown specifically rescues the mutant CHMP2B labelled structures, we quantified GFP-LAP CHMP2B^Intron5^ localization following *Tmem106b* ASO treatment. No difference was seen in the size or number of GFP-LAP CHMP2B^Intron5^ puncta following knockdown of *Tmem106b* (Fig. 8B and C), nor in the number of motile GFP-LAP CHMP2B^Intron5^ structures (Fig. 8D), indicating that the rescue of LysoTracker® trafficking and dendritic branching occurs via a general increase in trafficking. This is supported by the observations that the localization of TMEM106B is not altered in mutant CHMP2B neurons (Supplementary Fig. 8), and that *Tmem106b* ASO treatment does not enhance the ability of mutant CHMP2B to recruit VPS4 (Supplementary Fig. 9). These data indicate that reduction of TMEM106B may be a potential therapeutic for the treatment of early lysosomal trafficking defects more broadly than mutant CHMP2B-FTD.

## Discussion

Our data show for the first time that the FTD-causing C-terminal truncation of CHMP2B results in dendritic endolysosomal trafficking defects. Strikingly, we found that this trafficking defect could be rescued by the knockdown of the FTD risk factor *Tmem106b*. We also found that the expression of endogenous levels of mutant CHMP2B results in a reduction in LAMP2 structures at the neuronal soma. Enlarged endosomes have previously been detected in patient fibroblasts and cortex (Urwin *et al.* 2010), and our data in primary cortical cultures now indicates endolysosomal alterations occur as an early event within neurons in CHMP2B-FTD.

The reduction in LAMP2 structures could be due to several possibilities including (i) specifically reduced retrograde transport; (ii) reduced delivery of LAMP2 to lysosomes; (iii) reduced lysosome biogenesis; or (iv) reduced lysosome maturation. Our data indicate that the reduction is unlikely to be linked to the reduced trafficking we observe, as both retrograde and anterograde are equally affected by mutant CHMP2B, indicating a further distinct role of mutant CHMP2B at the lysosome.

FRAP analysis showed that mutant CHMP2B is stably localized to stationary endolysosomal structures. As expected, mutant CHMP2B containing endolysosomes were no longer able to recruit VPS4, which is consistent with a C-terminal CHMP2B missense mutation also reducing VPS4A binding (Han *et al.*, 2012). We propose a model whereby the incorporation of mutant CHMP2B into the forming intra-luminal vesicle arrests intra-luminal vesicle formation, prevents recruitment of VPS4, and renders the maturing endolysosome unable to travel any further along the neurite.

We found that at early stages in disease, at time points when endolysososmal disequilibrium is well established, no defects in the autophagosomal system were detected. Previous studies have reported autophagosomal defects following overexpression of mutant CHMP2B in both cell lines (Filimonenko *et al.*, 2007) and in primary cortical cultures (Lee *et al.*, 2007; Lee and Gao, 2009). Conversely, in a separate study neither enlarged endosomes nor autophagosome accumulation was observed when CHMP2B^Intron5^ or CHMP2B^Δ10^ were expressed in primary hippocampal neurons (Belly *et al.*, 2010). We did not detect any changes in autophagosomes in either mutant CHMP2B primary cultures, or in mutant CHMP2B mice up to 6 months of age. This indicates that autophagic defects occur downstream of the initial endolysosomal defects, but could still contribute to disease pathogenesis. For instance, functional multivesicular bodies are known to be necessary for the autophagy of protein aggregates associated with neurodegenerative conditions (Filimonenko *et al.* 2007).

Engorged stationary multivesicular bodies may be unable to fuse with either lysosomes or autophagosomes such that both pathways are ultimately affected. This could be caused by steric hindrance of fusion caused by one of two mechanisms. The shallow curvature of a bloated multivesicular body may interfere with tethering of late endosomes to lysosomes/autophagosomes. Alternatively, abnormal membrane dilution of SNARE complexes may affect fusion. Indeed, cholesterol accumulation in lysosomal storage disorders is known to impair SNARE function by sequestering SNAREs in aberrant spatial organizations, thus reducing the ability of endosomes to fuse with lysosomes (Fraldi *et al.*, 2010). In line with this possibility, we have previously reported that mutant CHMP2B can cause impaired fusion of endosomes and lysosomes in non-neuronal cells (Metcalf and Isaacs, 2010; Urwin *et al.*, 2010).

In addition to dendritic endolysosomal trafficking defects, we also report increased dendritic branching, specifically in distal dendrites, in mutant CHMP2B primary cortical neurons. This is in contrast to two previous studies, in which mutant CHMP2B over-expression led to reduced dendritic branching (Lee *et al.*, 2007; Belly *et al.*, 2010). The difference is likely due to the physiological expression of mutant CHMP2B in our system and highlights the importance of using physiological levels of proteins when investigating the endosomal system. Interestingly, loss of function of ESCRTs has been shown to increase dendritic branching in *Drosophila* larval sensory neurons (Sweeney *et al.*, 2006), potentially due to decreased dendritic pruning (Zhang *et al.*, 2014; Loncle *et al.*, 2015). Therefore the increased dendritic branching we observe is consistent with a role for ESCRTs in this process. It also indicates that in addition to the gain of function we have shown is necessary for causing neurodegenerative phenotypes (Ghazi-Noori *et al.*, 2012), mutant CHMP2B may also inhibit ESCRT function in some contexts.

Numerous lines of evidence now point to endolysosomal defects as a common event in FTD. For instance, while heterozygous mutations in *GRN* causes FTD, rare homozygous mutations in *GRN* have been found to cause neuronal ceroid lipofuscinosis (NCL), a lysosomal storage disorder (Smith *et al.*, 2012). Indeed, *Grn* knockout mice and *GRN* patients have features of both FTD and NCL, including increased levels of cathepsin D, LAMP1, saposin D and SCMAS (Gotzl *et al.*, 2014), and increased lipofuscinosis (Ward *et al.*, 2017). The ultimate downstream consequence of early endolysosomal trafficking defects in CHMP2B-FTD are the occurrence of large autofluorescent aggregates in both patients and mouse brain that are reminiscent of lysosomal storage pathology (Clayton *et al.*, 2015). Several studies have also shown that loss of function of C9orf72 can affect endosomal trafficking (O’Rourke *et al.*, 2016; Farg *et al.*, 2017; Shi *et al.*, 2018), which could contribute to causing FTD and ALS in concert with gain-of-function mechanisms (Balendra and Isaacs, 2018). Interestingly, knockdown of TDP-43 was recently shown to reduce the number and motility of dendritic recycling endosomes (Schwenk *et al.*, 2016). Finally, *TMEM106B*, the most well recognized risk factor for FTD (Van Deerlin *et al.*, 2010; Finch *et al.*, 2011; van der Zee and Van Broeckhoven, 2011; Gallagher *et al.*, 2014; van Blitterswijk *et al.*, 2014), is localized to late endosomes and lysosomes (Chen-Plotkin *et al.*, 2012; Lang *et al.*, 2012; Brady *et al.*, 2013). Altered risk associates with a coding variant in *TMEM106B*, with the risk allele degraded more slowly than the protective allele, leading to higher protein levels of TMEM106B (Nicholson *et al.*, 2013). A recent study identified a distinct risk variant as the likely causal variant and showed it led to increased *TMEM106B* RNA levels, via alteration of chromatin structure (Gallagher *et al.*, 2017). The common feature of both studies is that increased TMEM106B levels increase risk for FTD. Increasing TMEM106B levels by over-expression inhibits lysosomal transport (Stagi *et al.* 2014), which is consistent with our findings that an FTD-causing mutation in CHMP2B also decreases lysosomal transport.

As knockdown of *Tmem106b* conversely increases lysosomal dendritic traffic (Schwenk *et al.*, 2014), we used this approach to try to rescue the mutant CHMP2B-induced trafficking defect. Strikingly, ASO-mediated knockdown of *Tmem106b* rescued the trafficking defects we report in our mutant CHMP2B neurons. Importantly, this led to a functional rescue of the associated mutant CHMP2B-induced dendritic branching defect. TMEM106B shows a partial overlap with CHMP2B-positive structures (Jun *et al.*, 2015) suggesting the two may be present on the same endolysosomes. However, we did not observe any functional rescue of mutant CHMP2B-positive immobilized structures upon knockdown of *Tmem106b*. Instead we hypothesize that *Tmem106b* knockdown may serve to upregulate lysosomal trafficking by mobilizing extra endolysosomal vesicles from a pool that are ordinarily stationary. Interestingly, we did not observe dramatic changes in lysosomal trafficking or dendritic branching when the *Tmem106b* ASOs were applied to control neurons. This is in contrast to previous reports, which show much stronger effects in control neurons using shRNAs directed against *Tmem106b*. One explanation may be the greater level of knockdown in those studies. This suggests that a lower level of knockdown may be sufficient to reduce trafficking and branching defects when neurons are under stress or already impaired, such as expression of mutant CHMP2B, but that greater knockdown is required for effects under basal conditions. This may also indicate that there is an optimal level of knockdown to improve compromised neurons without affecting healthy neurons.

Previously, alterations in dendritic trafficking dynamics have been found to influence dendritic arborization in several studies. Numerous *Drosophila* mutant models have shown a link between altered dendritic branching and (i) motor proteins; (ii) endocytic pathways; and (iii) local translation (for a comprehensive review see Jan and Jan, 2010). Altered dendritic trafficking may influence local signalling, which affects neuronal arborization. Indeed, expression of constitutively active Rab11 increases the localization of TrkB to dendrites, thereby increasing local TrkB signalling and regulating arborization (Lazo *et al.*, 2013). Thus *Tmem106b* knockdown may mobilize a pool of endolysosomal structures sufficient to restore appropriate dendritic signalling pathways in mutant CHMP2B neurons.

This indicates a more general effect on trafficking that could be broadly beneficial for restoring endolysosomal trafficking impairment. Consistent with this possibility, TMEM106B interacts with MAP6 to deliver a stop signal to endolysosomal vesicles (Schwenk *et al.*, 2014). Knockdown of *Tmem106b* in our mutant CHMP2B neurons may remove this endolysosomal stop signal, and result in greater motility of the remaining endolysosomal pool, thereby compensating for the trafficking defects we observe in our mutant CHMP2B neurons. Interestingly, it was recently reported that knockout of *Tmem106b* in mice can rescue lysosomal defects caused by loss of *Grn* (Klein *et al.*, 2017), due to *Tmem106b* knockout and *Grn* knockout having opposing effects on lysosomal function, although very limited benefit was observed in a second study (Arrant *et al.*, 2018). Remarkably, we also observe that *Tmem106b* knockdown has opposing effects to another FTD gene, mutant *CHMP2B*, in both endolysosomal trafficking and dendritic branching.

We used ASO treatment to knockdown *Tmem106b* in our mutant CHMP2B neuronal cultures. ASOs represent a promising therapeutic to treat neurodegenerative diseases, as they are highly specific and well tolerated (Evers *et al.*, 2015). ASO treatment for spinal muscular atrophy was recently approved following successful clinical trials (Finkel *et al.*, 2016) and ASOs are also in clinical trial for SOD1 ALS (Miller *et al.*, 2013). ASOs have also been used to successfully alleviate symptoms in various model systems of ALS, including *C9orf72* patient-induced pluripotent stem cell (iPSC) neurons, and TDP-43 and *C9orf72* mouse models (Donnelly *et al.*, 2013; Sareen *et al.*, 2013; Jiang *et al.*, 2016; Becker *et al.*, 2017; Scoles *et al.*, 2017), indicating their therapeutic potential.

With an increasing body of evidence now linking FTD causative mutations to dysfunction of the endosomal and lysosomal system, it is becoming apparent that there is a susceptibility of cortical neurons to endolysosomal defects. Exactly why cortical neurons are so susceptible to endolysosomal defects is unclear. However, this common disease mechanism raises the exciting possibility of treatment targeting intracellular trafficking as a potentially widely applicable therapy for FTD.

## Supplementary Material

Supplementary Data

## Abbreviations

ALS: amyotrophic lateral sclerosis
ASO: antisense oligonucleotide
ESCRT-III: endosomal sorting complex required for transport-III
FTD: frontotemporal dementia

## References

[awy284-B71] Arrant AE, Nicholson AM, Zhou X, Rademakers R, Roberson ED Partial Tmem106b reduction does not correct abnormalities due to progranulin haploinsufficiency. Mol Neurodegener 2018; 13: 32. 29929528 10.1186/s13024-018-0264-6 PMC6013889

[awy284-B1] Balendra R, Isaacs AM C9orf72-mediated ALS and FTD: multiple pathways to disease. Nat Rev Neurol 2018; 14: 544–58. 30120348 10.1038/s41582-018-0047-2 PMC6417666

[awy284-B2] Becker LA, Huang B, Bieri G, Ma R, Knowles DA, Jafar-Nejad P, et al Therapeutic reduction of ataxin-2 extends lifespan and reduces pathology in TDP-43 mice. Nature 2017; 544: 367–71. 28405022 10.1038/nature22038 PMC5642042

[awy284-B3] Belly A, Bodon G, Blot B, Bouron A, Sadoul R, Goldberg Y CHMP2B mutants linked to frontotemporal dementia impair maturation of dendritic spines. J Cell Sci 2010; 123: 2943–54. 20699355 10.1242/jcs.068817 PMC3013364

[awy284-B4] Bennion CJ, Pickering-Brown SM Pathogenesis/genetics of frontotemporal dementia and how it relates to ALS. Exp Neurol 2014; 262 (Pt B): 84–90. 24915640 10.1016/j.expneurol.2014.06.001 PMC4221591

[awy284-B5] Bishop N, Woodman P ATPase-defective mammalian VPS4 localizes to aberrant endosomes and impairs cholesterol trafficking. Mol Biol Cell 2000; 11: 227–39. 10637304 10.1091/mbc.11.1.227 PMC14770

[awy284-B6] Brady OA, Zheng Y, Murphy K, Huang M, Hu F The frontotemporal lobar degeneration risk factor, TMEM106B, regulates lysosomal morphology and function. Hum Mol Genet 2013; 22: 685–95. 23136129 10.1093/hmg/dds475 PMC3554197

[awy284-B7] Carlton JG, Martin-Serrano J Parallels between cytokinesis and retroviral budding: a role for the ESCRT machinery. Science 2007; 316: 1908–12. 17556548 10.1126/science.1143422

[awy284-B8] Chen-Plotkin AS, Unger TL, Gallagher MD, Bill E, Kwong LK, Volpicelli-Daley L, et al TMEM106B, the risk gene for frontotemporal dementia, is regulated by the microRNA-132/212 cluster and affects progranulin pathways. J Neurosci 2012; 32: 11213–27. 22895706 10.1523/JNEUROSCI.0521-12.2012 PMC3446826

[awy284-B9] Clayton EL, Mizielinska S, Edgar JR, Nielsen TT, Marshall S, Norona FE, et al Frontotemporal dementia caused by CHMP2B mutation is characterised by neuronal lysosomal storage pathology. Acta Neuropathol 2015; 130: 511–23. 26358247 10.1007/s00401-015-1475-3 PMC4575387

[awy284-B10] Donnelly CJ, Zhang PW, Pham JT, Haeusler AR, Mistry NA, Vidensky S, et al RNA toxicity from the ALS/FTD C9ORF72 expansion is mitigated by antisense intervention. Neuron 2013; 80: 415–28. 24139042 10.1016/j.neuron.2013.10.015 PMC4098943

[awy284-B11] Evers MM, Toonen LJ, van Roon-Mom WM Antisense oligonucleotides in therapy for neurodegenerative disorders. Adv Drug Deliv Rev 2015; 87: 90–103. 25797014 10.1016/j.addr.2015.03.008

[awy284-B12] Farg MA, Sundaramoorthy V, Sultana JM, Yang S, Atkinson RA, Levina V, et al C9ORF72, implicated in amytrophic lateral sclerosis and frontotemporal dementia, regulates endosomal trafficking. Hum Mol Genet 2017; 26: 4093–4. 28973528 10.1093/hmg/ddx309 PMC5886132

[awy284-B13] Filimonenko M, Stuffers S, Raiborg C, Yamamoto A, Malerød L, Fisher EM, et al Functional multivesicular bodies are required for autophagic clearance of protein aggregates associated with neurodegenerative disease. J Cell Biol 2007; 179: 485–500. 17984323 10.1083/jcb.200702115 PMC2064794

[awy284-B14] Finch N, Carrasquillo MM, Baker M, Rutherford NJ, Coppola G, Dejesus-Hernandez M, et al TMEM106B regulates progranulin levels and the penetrance of FTLD in GRN mutation carriers. Neurology 2011; 76: 467–74. 21178100 10.1212/WNL.0b013e31820a0e3b PMC3034409

[awy284-B15] Finkel RS, Chiriboga CA, Vajsar J, Day JW, Montes J, De Vivo DC, et al Treatment of infantile-onset spinal muscular atrophy with nusinersen: a phase 2, open-label, dose-escalation study. Lancet 2016; 388: 3017–26. 27939059 10.1016/S0140-6736(16)31408-8

[awy284-B16] Fraldi A, Annunziata F, Lombardi A, Kaiser HJ, Medina DL, Spampanato C, et al Lysosomal fusion and SNARE function are impaired by cholesterol accumulation in lysosomal storage disorders. EMBO J 2010; 29: 3607–20. 20871593 10.1038/emboj.2010.237 PMC2982760

[awy284-B17] Gallagher MD, Posavi M, Huang P, Unger TL, Berlyand Y, Gruenewald AL, et al A dementia-associated risk variant near TMEM106B alters chromatin architecture and gene expression. Am J Hum Genet 2017; 101: 643–63. 29056226 10.1016/j.ajhg.2017.09.004 PMC5673619

[awy284-B18] Gallagher MD, Suh E, Grossman M, Elman L, McCluskey L, Van Swieten JC, et al TMEM106B is a genetic modifier of frontotemporal lobar degeneration with C9orf72 hexanucleotide repeat expansions. Acta Neuropathol 2014; 127: 407–18. 24442578 10.1007/s00401-013-1239-x PMC4003885

[awy284-B19] Gascon E, Lynch K, Ruan H, Almeida S, Verheyden JM, Seeley WW, et al Alterations in microRNA-124 and AMPA receptors contribute to social behavioral deficits in frontotemporal dementia. Nat Med 2014; 20: 1444–51. 25401692 10.1038/nm.3717 PMC4257887

[awy284-B20] Ghazi-Noori S, Froud KE, Mizielinska S, Powell C, Smidak M, Fernandez de Marco M, et al Progressive neuronal inclusion formation and axonal degeneration in CHMP2B mutant transgenic mice. Brain 2012; 135: 819–32. 22366797 10.1093/brain/aws006

[awy284-B21] Gijselinck I, Van MS, van der Zee J, Sieben A, Philtjens S, Heeman B, et al Loss of TBK1 is a frequent cause of frontotemporal dementia in a Belgian cohort. Neurology 2015; 85: 2116–25. 26581300 10.1212/WNL.0000000000002220 PMC4691687

[awy284-B22] Gotzl JK, Mori K, Damme M, Fellerer K, Tahirovic S, Kleinberger G, et al Common pathobiochemical hallmarks of progranulin-associated frontotemporal lobar degeneration and neuronal ceroid lipofuscinosis. Acta Neuropathol 2014; 127: 845–60. 24619111 10.1007/s00401-014-1262-6

[awy284-B23] Han JH, Ryu HH, Jun MH, Jang DJ, Lee JA The functional analysis of the CHMP2B missense mutation associated with neurodegenerative diseases in the endo-lysosomal pathway. Biochem Biophys Res Commun 2012; 421: 544–9. 22521643 10.1016/j.bbrc.2012.04.041

[awy284-B24] Harvey RJ, Skelton-Robinson M, Rossor MN The prevalence and causes of dementia in people under the age of 65 years. J Neurol Neurosurg Psychiatry 2003; 74: 1206–9. 12933919 10.1136/jnnp.74.9.1206 PMC1738690

[awy284-B25] Jan YN, Jan LY Branching out: mechanisms of dendritic arborization. Nat Rev Neurosci 2010; 11: 316–28. 20404840 10.1038/nrn2836 PMC3079328

[awy284-B26] Jiang J, Zhu Q, Gendron TF, Saberi S, McAlonis-Downes M, Seelman A, et al Gain of toxicity from ALS/FTD-linked repeat expansions in C9ORF72 is alleviated by antisense oligonucleotides targeting GGGGCC-containing RNAs. Neuron 2016; 90: 535–50. 27112497 10.1016/j.neuron.2016.04.006 PMC4860075

[awy284-B27] Jun MH, Han JH, Lee YK, Jang DJ, Kaang BK, Lee JA TMEM106B, a frontotemporal lobar dementia (FTLD) modifier, associates with FTD-3-linked CHMP2B, a complex of ESCRT-III. Mol Brain 2015; 8: 85. 26651479 10.1186/s13041-015-0177-z PMC4676093

[awy284-B28] Klein ZA, Takahashi H, Ma M, Stagi M, Zhou M, Lam TT, et al Loss of TMEM106B ameliorates lysosomal and frontotemporal dementia-related phenotypes in progranulin-deficient mice. Neuron 2017; 95: 281–96. 28728022 10.1016/j.neuron.2017.06.026 PMC5558861

[awy284-B29] Lang CM, Fellerer K, Schwenk BM, Kuhn PH, Kremmer E, Edbauer D, et al Membrane orientation and subcellular localization of transmembrane protein 106B (TMEM106B), a major risk factor for frontotemporal lobar degeneration. J Biol Chem 2012; 287: 19355–65. 22511793 10.1074/jbc.M112.365098 PMC3365973

[awy284-B30] Lata S, Schoehn G, Jain A, Pires R, Piehler J, Gottlinger HG, et al Helical structures of ESCRT-III are disassembled by VPS4. Science 2008; 321: 1354–7. 18687924 10.1126/science.1161070 PMC2758909

[awy284-B31] Lazo OM, Gonzalez A, Ascano M, Kuruvilla R, Couve A, Bronfman FC BDNF regulates Rab11-mediated recycling endosome dynamics to induce dendritic branching. J Neurosci 2013; 33: 6112–22. 23554492 10.1523/JNEUROSCI.4630-12.2013 PMC3684039

[awy284-B32] Le Ber I, De SA, Millecamps S, Camuzat A, Caroppo P, Couratier P, et al TBK1 mutation frequencies in French frontotemporal dementia and amyotrophic lateral sclerosis cohorts. Neurobiol Aging 2015; 36: 3116–18. 10.1016/j.neurobiolaging.2015.08.009 26476236

[awy284-B33] Lee JA, Beigneux A, Ahmad ST, Young SG, Gao FB ESCRT-III dysfunction causes autophagosome accumulation and neurodegeneration. Curr Biol 2007; 17: 1561–7. 17683935 10.1016/j.cub.2007.07.029

[awy284-B34] Lee JA, Gao FB Inhibition of autophagy induction delays neuronal cell loss caused by dysfunctional ESCRT-III in frontotemporal dementia. J Neurosci 2009; 29: 8506–11. 19571141 10.1523/JNEUROSCI.0924-09.2009 PMC2726650

[awy284-B35] Lindquist SG, Braedgaard H, Svenstrup K, Isaacs AM, Nielsen JE Frontotemporal dementia linked to chromosome 3 (FTD-3)–current concepts and the detection of a previously unknown branch of the Danish FTD-3 family. Eur J Neurol 2008; 15: 667–70. 18484988 10.1111/j.1468-1331.2008.02144.x

[awy284-B36] Loncle N, Agromayor M, Martin-Serrano J, Williams DW An ESCRT module is required for neuron pruning. Sci Rep 2015; 5: 8461. 25676218 10.1038/srep08461 PMC4327575

[awy284-B37] McKhann GM, Albert MS, Grossman M, Miller B, Dickson D, Trojanowski JQ Clinical and pathological diagnosis of frontotemporal dementia: report of the Work Group on Frontotemporal Dementia and Pick ’s Disease. Arch Neurol 2001; 58: 1803–9. 11708987 10.1001/archneur.58.11.1803

[awy284-B38] Metcalf D, Isaacs AM The role of ESCRT proteins in fusion events involving lysosomes, endosomes and autophagosomes. Biochem Soc Trans 2010; 38: 1469–73. 21118109 10.1042/BST0381469

[awy284-B39] Mierzwa BE, Chiaruttini N, Redondo-Morata L, von Filseck JM, König J, Larios J, et al Dynamic subunit turnover in ESCRT-III assemblies is regulated by Vps4 to mediate membrane remodelling during cytokinesis. Nat Cell Biol 2017; 19: 787–98. 28604678 10.1038/ncb3559 PMC5493987

[awy284-B40] Miller TM, Pestronk A, David W, Rothstein J, Simpson E, Appel SH, et al An antisense oligonucleotide against SOD1 delivered intrathecally for patients with SOD1 familial amyotrophic lateral sclerosis: a phase 1, randomised, first-in-man study. Lancet Neurol 2013; 12: 435–42. 23541756 10.1016/S1474-4422(13)70061-9 PMC3712285

[awy284-B41] Mizushima N, Yamamoto A, Matsui M, Yoshimori T, Ohsumi Y In vivo analysis of autophagy in response to nutrient starvation using transgenic mice expressing a fluorescent autophagosome marker. Mol Biol Cell 2004; 15: 1101–11. 14699058 10.1091/mbc.E03-09-0704 PMC363084

[awy284-B42] Morita E, Sundquist WI Retrovirus budding. Annu Rev Cell Dev Biol 2004; 20: 395–425. 15473846 10.1146/annurev.cellbio.20.010403.102350

[awy284-B43] Neary D, Snowden JS, Gustafson L, Passant U, Stuss D, Black S, et al Frontotemporal lobar degeneration: a consensus on clinical diagnostic criteria. Neurology 1998; 51: 1546–54. 9855500 10.1212/wnl.51.6.1546

[awy284-B44] Nicholson AM, Finch NA, Wojtas A, Baker MC, Perkerson RB 3rd, Castanedes-Casey M, et al TMEM106B p.T185S regulates TMEM106B protein levels: implications for frontotemporal dementia. J Neurochem 2013; 126: 781–91. 23742080 10.1111/jnc.12329 PMC3766501

[awy284-B45] O’Rourke JG, Bogdanik L, Yanez A, Lall D, Wolf AJ, Muhammad AK, et al C9orf72 is required for proper macrophage and microglial function in mice. Science 2016; 351: 1324–9. 26989253 10.1126/science.aaf1064 PMC5120541

[awy284-B46] Olmos Y, Hodgson L, Mantell J, Verkade P, Carlton JG ESCRT-III controls nuclear envelope reformation. Nature 2015; 522: 236–9. 26040713 10.1038/nature14503 PMC4471131

[awy284-B47] Pottier C, Bieniek KF, Finch N, van de Vorst M, Baker M, Perkersen R, et al Whole-genome sequencing reveals important role for TBK1 and OPTN mutations in frontotemporal lobar degeneration without motor neuron disease. Acta Neuropathol 2015; 130: 77–92. 25943890 10.1007/s00401-015-1436-x PMC4470809

[awy284-B48] Raiborg C, Stenmark H The ESCRT machinery in endosomal sorting of ubiquitylated membrane proteins. Nature 2009; 458: 445–52. 19325624 10.1038/nature07961

[awy284-B49] Ratnavalli E, Brayne C, Dawson K, Hodges JR The prevalence of frontotemporal dementia. Neurology 2002; 58: 1615–21. 12058088 10.1212/wnl.58.11.1615

[awy284-B50] Rohrer JD, Warren JD Phenotypic signatures of genetic frontotemporal dementia. Curr Opin Neurol 2011; 24: 542–9. 21986680 10.1097/WCO.0b013e32834cd442

[awy284-B51] Sareen D, O’Rourke JG, Meera P, Muhammad AK, Grant S, Simpkinson M, et al Targeting RNA foci in iPSC-derived motor neurons from ALS patients with a C9ORF72 repeat expansion. Sci Transl Med 2013; 5: 208ra149. 10.1126/scitranslmed.3007529 PMC4090945 24154603

[awy284-B52] Schuh AL, Audhya A The ESCRT machinery: from the plasma membrane to endosomes and back again. Crit Rev Biochem Mol Biol 2014; 49: 242–61. 24456136 10.3109/10409238.2014.881777 PMC4381963

[awy284-B53] Schwenk BM, Hartmann H, Serdaroglu A, Schludi MH, Hornburg D, Meissner F, et al TDP-43 loss of function inhibits endosomal trafficking and alters trophic signaling in neurons. EMBO J 2016; 35: 2350–70. 27621269 10.15252/embj.201694221 PMC5090220

[awy284-B54] Schwenk BM, Lang CM, Hogl S, Tahirovic S, Orozco D, Rentzsch K, et al The FTLD risk factor TMEM106B and MAP6 control dendritic trafficking of lysosomes. EMBO J 2014; 33: 450–67. 24357581 10.1002/embj.201385857 PMC3989627

[awy284-B55] Scoles DR, Meera P, Schneider MD, Paul S, Dansithong W, Figueroa KP, et al Antisense oligonucleotide therapy for spinocerebellar ataxia type 2. Nature 2017; 544: 362–6. 28405024 10.1038/nature22044 PMC6625650

[awy284-B56] Shi Y, Lin S, Staats KA, Li Y, Chang WH, Hung ST, et al Haploinsufficiency leads to neurodegeneration in C9ORF72 ALS/FTD human induced motor neurons. Nat Med 2018; 24: 313–25. 29400714 10.1038/nm.4490 PMC6112156

[awy284-B57] Skibinski G, Parkinson NJ, Brown JM, Chakrabarti L, Lloyd SL, Hummerich H, et al Mutations in the endosomal ESCRTIII-complex subunit CHMP2B in frontotemporal dementia. Nat Genet 2005; 37: 806–8. 16041373 10.1038/ng1609

[awy284-B58] Smith KR, Damiano J, Franceschetti S, Carpenter S, Canafoglia L, Morbin M, et al Strikingly different clinicopathological phenotypes determined by progranulin-mutation dosage. Am J Hum Genet 2012; 90: 1102–7. 22608501 10.1016/j.ajhg.2012.04.021 PMC3370276

[awy284-B59] Stagi M, Klein ZA, Gould TJ, Bewersdorf J, Strittmatter SM Lysosome size, motility and stress response regulated by fronto-temporal dementia modifier TMEM106B. Mol Cell Neurosci 2014; 61: 226–40. 25066864 10.1016/j.mcn.2014.07.006 PMC4145808

[awy284-B60] Stuchell-Brereton MD, Skalicky JJ, Kieffer C, Karren MA, Ghaffarian S, Sundquist WI ESCRT-III recognition by VPS4 ATPases. Nature 2007; 449: 740–4. 17928862 10.1038/nature06172

[awy284-B72] Swayze EE, Siwkowski AM, Wancewicz EV, Migawa MT, Wyrzykiewicz TK, Hung G, et al Antisense oligonucleotides containing locked nucleic acid improve potency but cause significant hepatotoxicity in animals. Nucleic Acids Res 2007; 35: 687–700. 17182632 10.1093/nar/gkl1071 PMC1802611

[awy284-B61] Sweeney NT, Brenman JE, Jan YN, Gao FB The coiled-coil protein shrub controls neuronal morphogenesis in Drosophila. Curr Biol 2006; 16: 1006–11. 16713958 10.1016/j.cub.2006.03.067

[awy284-B62] Urwin H, Authier A, Nielsen JE, Metcalf D, Powell C, Froud K, et al Disruption of endocytic trafficking in frontotemporal dementia with CHMP2B mutations. Hum Mol Genet 2010; 19: 2228–38. 20223751 10.1093/hmg/ddq100 PMC2865375

[awy284-B63] van Blitterswijk M, Mullen B, Nicholson AM, Bieniek KF, Heckman MG, Baker MC, et al TMEM106B protects C9ORF72 expansion carriers against frontotemporal dementia. Acta Neuropathol 2014; 127: 397–406. 24385136 10.1007/s00401-013-1240-4 PMC3944829

[awy284-B64] Van Deerlin VM, Sleiman PM, Martinez-Lage M, Chen-Plotkin A, Wang LS, Graff-Radford NR, et al Common variants at 7p21 are associated with frontotemporal lobar degeneration with TDP-43 inclusions. Nat Genet 2010; 42: 234–9. 20154673 10.1038/ng.536 PMC2828525

[awy284-B65] van der Zee J, Gijselinck I, Van Mossevelde S, Perrone F, Dillen L, Heeman B, et al TBK1 mutation spectrum in an extended european patient cohort with frontotemporal dementia and amyotrophic lateral sclerosis. Hum Mutat 2017; 38: 297–309. 28008748 10.1002/humu.23161 PMC5324646

[awy284-B66] van der Zee J, Van Broeckhoven C TMEM106B a novel risk factor for frontotemporal lobar degeneration. J Mol Neurosci 2011; 45: 516–521. 21614538 10.1007/s12031-011-9555-x PMC3207134

[awy284-B67] Vietri M, Schink KO, Campsteijn C, Wegner CS, Schultz SW, Christ L, et al Spastin and ESCRT-III coordinate mitotic spindle disassembly and nuclear envelope sealing. Nature 2015; 522: 231–35. 26040712 10.1038/nature14408

[awy284-B68] Ward ME, Chen R, Huang HY, Ludwig C, Telpoukhovskaia M, Taubes A, et al Individuals with progranulin haploinsufficiency exhibit features of neuronal ceroid lipofuscinosis. Sci Transl Med 2017; 9: eaah5642. 28404863 10.1126/scitranslmed.aah5642 PMC5526610

[awy284-B69] Wollert T, Wunder C, Lippincott-Schwartz J, Hurley JH Membrane scission by the ESCRT-III complex. Nature 2009; 458: 172–7. 19234443 10.1038/nature07836 PMC2743992

[awy284-B70] Zhang H, Wang Y, Wong JJ, Lim KL, Liou YC, Wang H, et al Endocytic pathways downregulate the L1-type cell adhesion molecule neuroglian to promote dendrite pruning in Drosophila. Dev Cell 2014; 30: 463–78. 25158855 10.1016/j.devcel.2014.06.014

