# Supplementary material for: Frontotemporal dementia causative CHMP2B impairs neuronal endolysosomal traffic-rescue by TMEM106B knockdown
Source: Brain. 2018 Nov 29;141(12):3428–42. doi: 10.1093/brain/awy284 (PMC6262218; doi:10.1093/brain/awy284)
Supplement: Supplementary Data [file awy284_suppl_data.pdf]

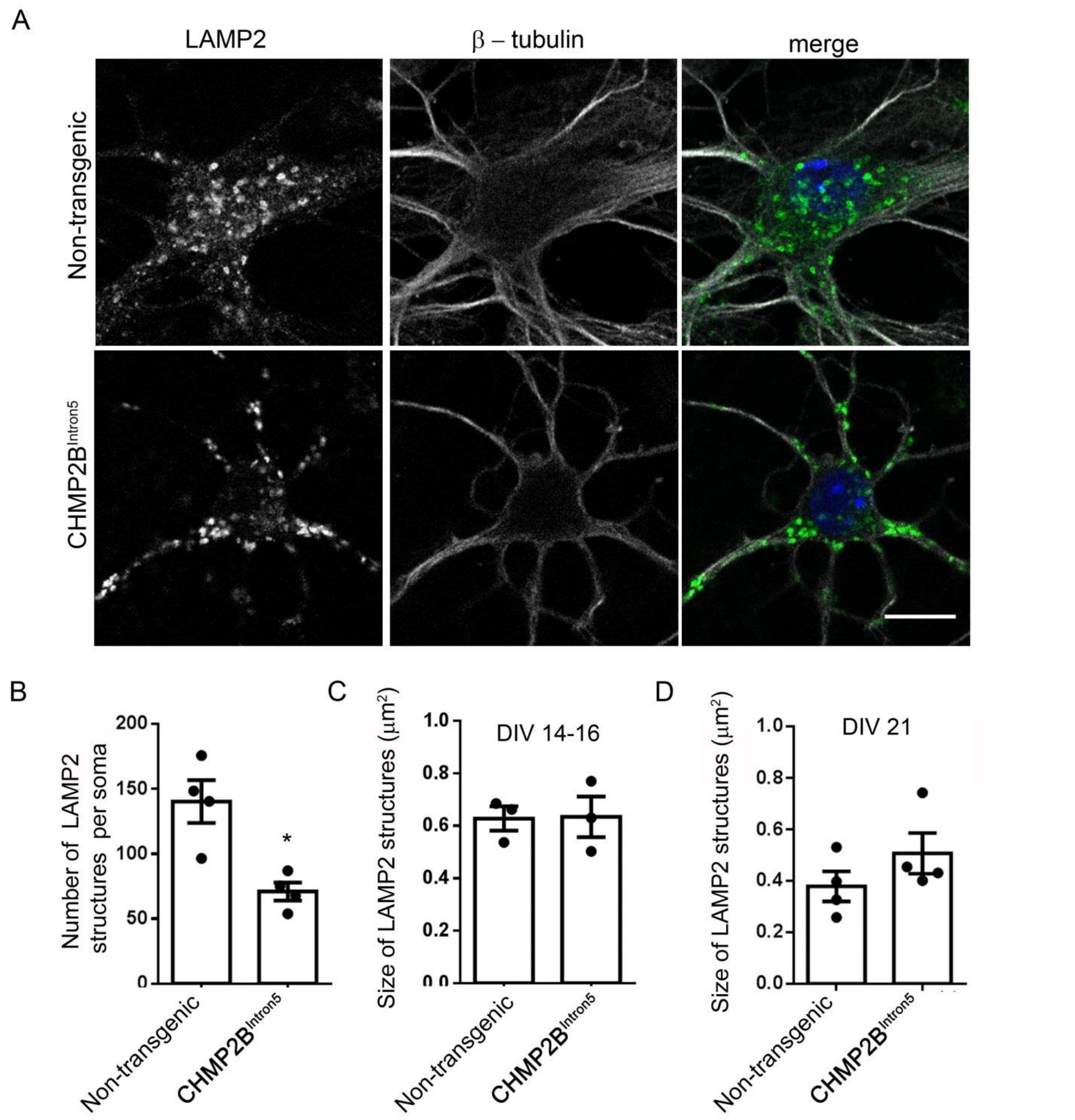

Supplementary Figure 1. LAMP2 staining in DIV 21 cortical neurons. (**A**) Representative images for LAMP2 staining in control and mutant CHMP2B neurons at 21 DIV. Scale bar 10 μm. (**B**) Quantification of the number of LAMP2 structures per soma. Unpaired t-test. * = $p < 0.05$, N=3 with 4-6 neurons per N. (**C**) Quantification of the size of LAMP2 structures in the soma of DIV 14-16 and (**D**) DIV 21 neurons.

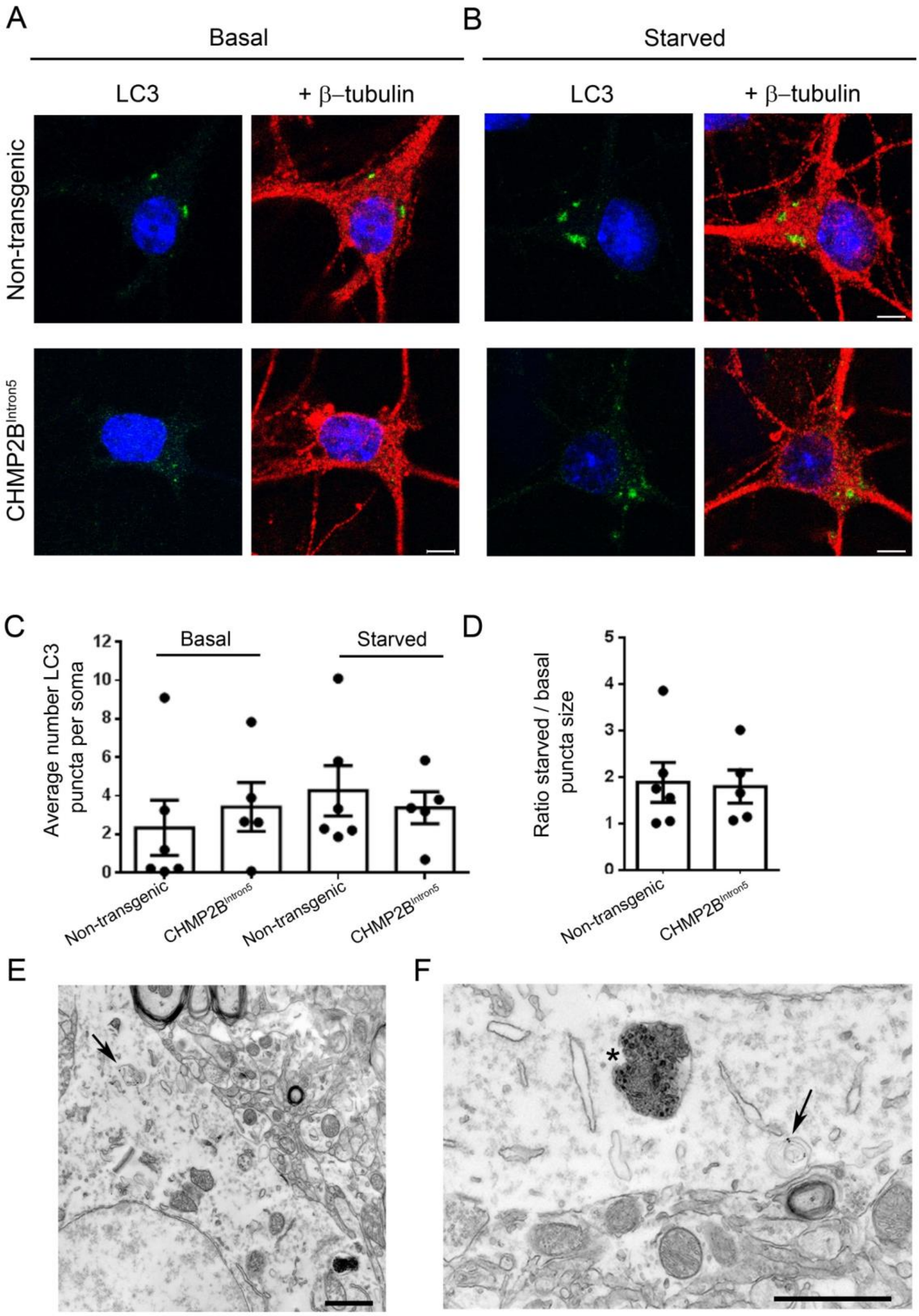

Supplementary Figure 2. No accumulation of autophagosomes in mutant CHMP2B primary cortical cultures or 6 month old mouse brain. (**A**) LC3 staining (green) in control non-transgenic (upper panels) and mutant CHMP2B (lower panels) DIV 12 – 14 cortical cultures neurons are labelled with β-tubulin (red) and nuclei with DAPI (blue). Representative images are shown from cultures under (**A**) basal conditions or (**B**) after 6 hours starvation. Scale bar 5 µm. (**C**) Quantification of the number of LC3 positive puncta per

neuron per condition. N= 5 for mutant CHMP2B, N = 6 for control with 8 -18 neurons per N. (**D**) Quantification of the ratio of the size of LC3 positive puncta measured under basal conditions to the size following starvation shows LC3 puncta increased in size after autophagy induction in both control and mutant CHMP2B neurons (**E, F**) Immuno-EM of 6 month sections from mutant CHMP2B GFP-LC3 mice. Sections were gold immuno-labelled with an antibody against GFP. Arrows indicates a GFP labelled autophagosome. Asterisk indicates a lysosomal storage inclusion, which is negative for GFP-LC3. Scale bars = 1 μm.

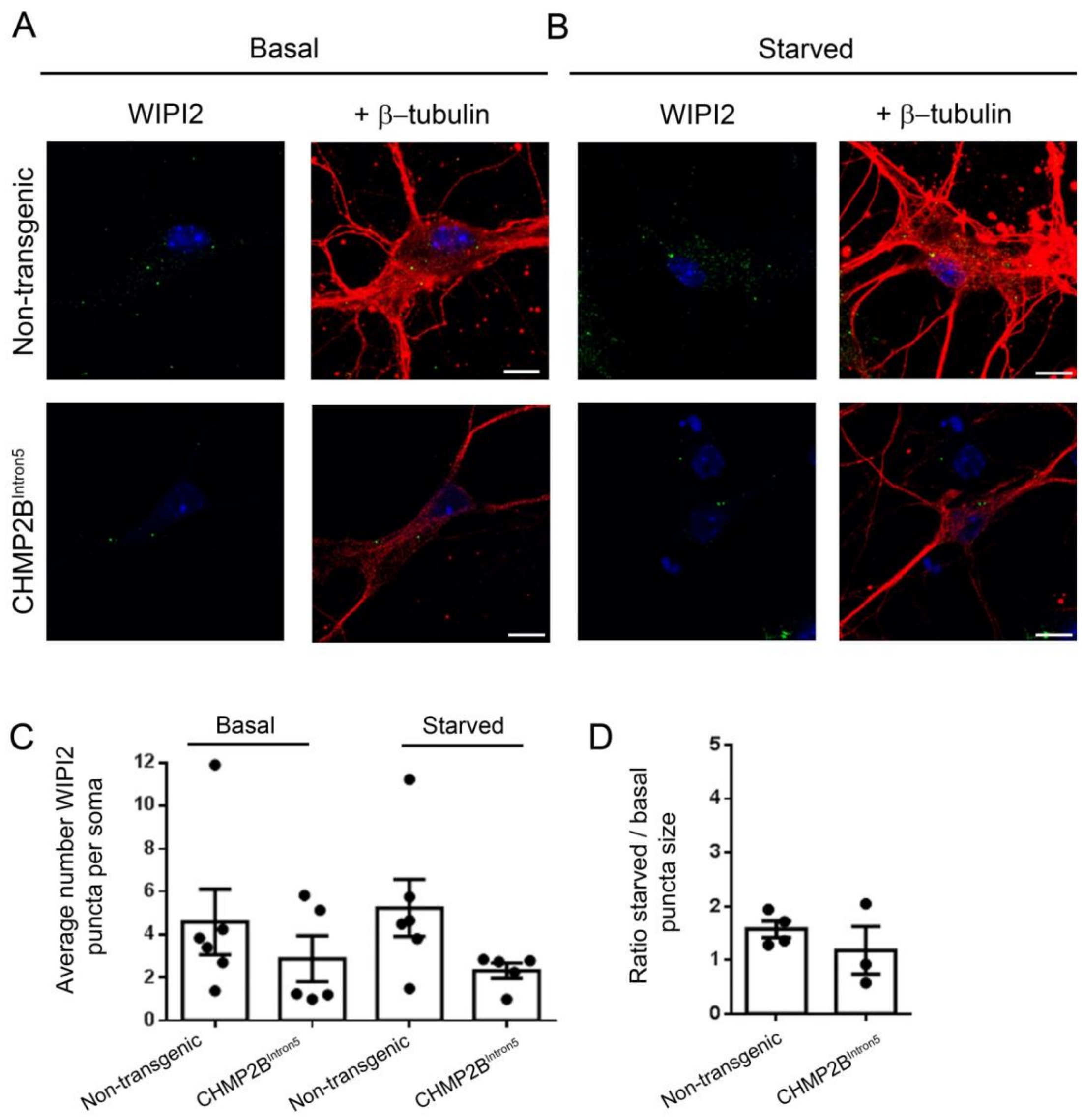

Supplementary Figure 3. WIPI2 staining in mutant CHMP2B primary cortical cultures. (**A**) WIPI2 staining in control non-transgenic (upper panels) and mutant CHMP2B (lower panels) DIV 12 – 14 cortical cultures. Representative images are shown from cultures under (**A**) basal conditions or (**B**) after 6 hours starvation. Green = WIPI2 staining, red = β-tubulin, and DAPI is blue. Scale bar 5 µm. (**C**) Quantification of the number of WIPI2 positive puncta per neuron per condition. N= 5 for mutant CHMP2B, N = 6 for control with 8 -18 neurons per N. (**D**) Quantification of the ratio of the size of WIPI2 positive puncta measured under basal conditions to the size following starvation in control and mutant CHMP2B neurons.

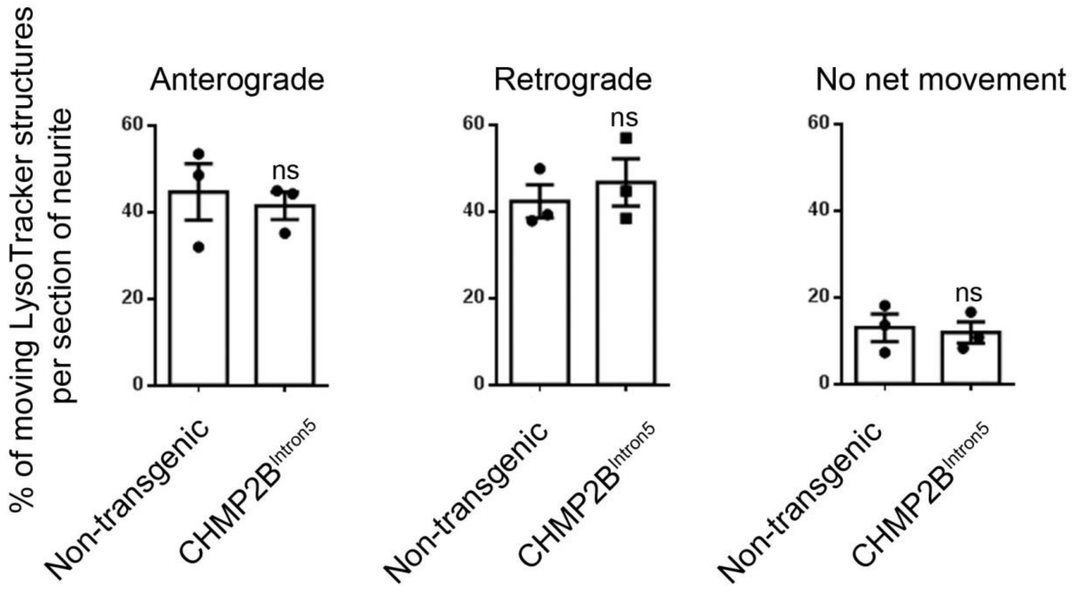

Supplementary Figure 4. Mutant CHMP2B impairs both anterograde and retrograde trafficking of LysoTracker structures. The proportion of all moving structures which moved in anterograde, retrograde or with no net movement (oscillating structures) are shown as labelled per genotype. No significant difference was seen in the directional trafficking of the pool of moving LysoTracker labelled structures as compared to controls. ns = not significant. Unpaired t-test. N = 3 with 3 -5 neurons per N.

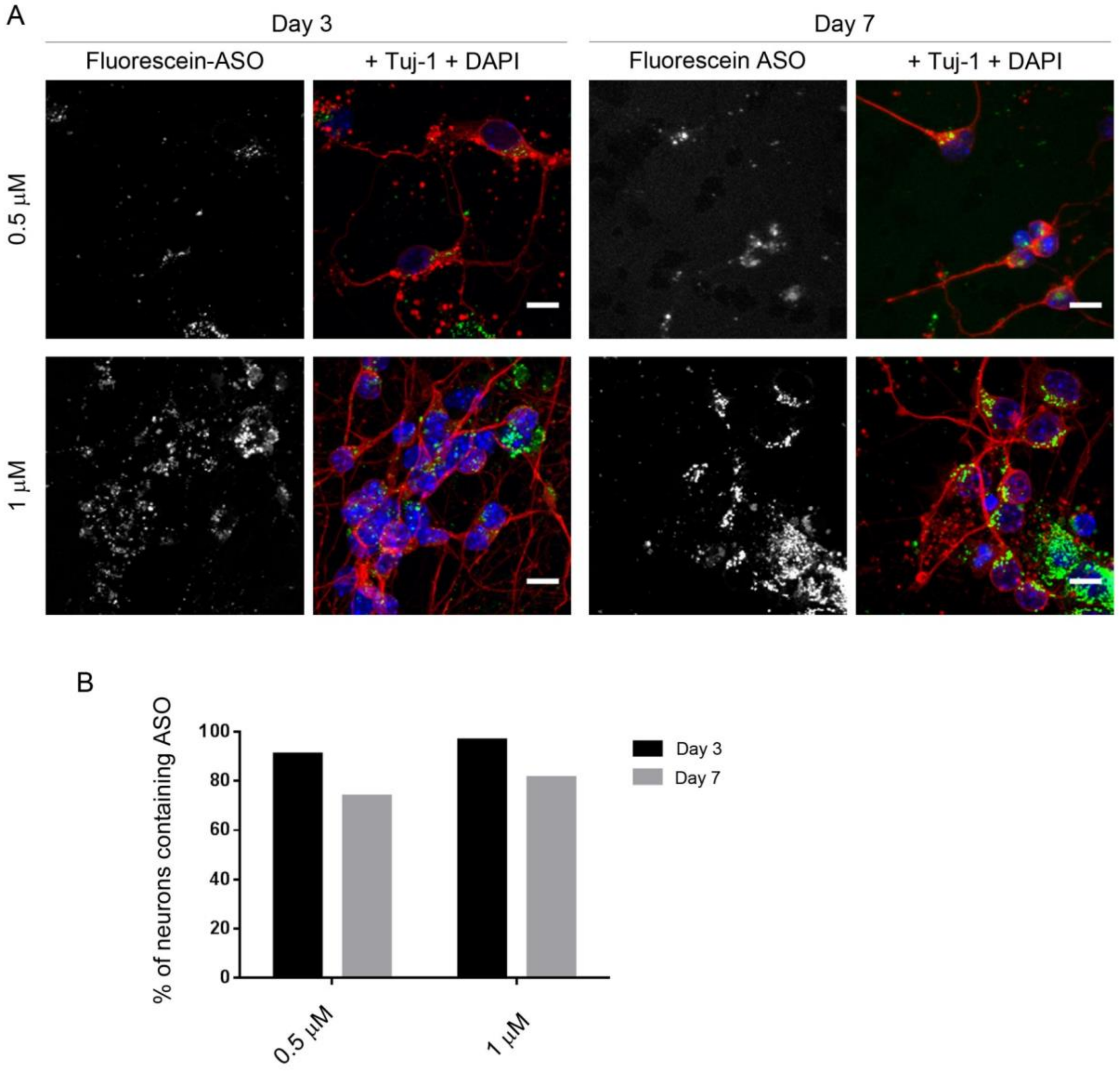

Supplementary Figure 5. Fluorescent ASO internalisation in primary cortical neurons. (**A**) Representative images of primary cortical cultures incubated with 0.5 or 1 µM fluorescein conjugated ASO at plating, and fixed at the indicated timepoints. Green = fluorescein-ASO, red = Tuj-1 and blue = DAPI. Scale bar = 10 µm. (B) 100 neurons (based on Tuj-1 staining) were scored for presence of fluorescent ASO at 3 and 7 days post ASO addition.

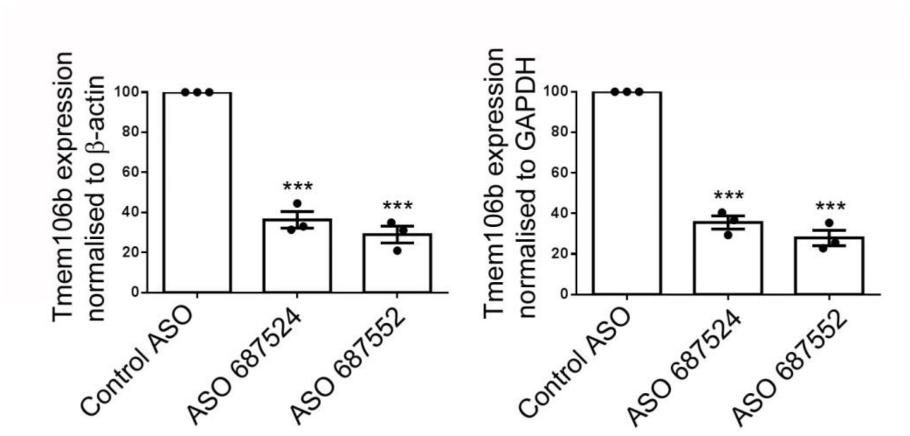

Supplementary Figure 6. Tmem106b knockdown after 7 days of ASO treatment. (**A**) Quantification of Tmem106b mRNA levels in mutant CHMP2B cortical cultures following 7 days of treatment with 5 µM of the indicated ASOs normalised to either ß-actin (on left) or GAPDH (on right), N = 3. *** = $p < 0.01$, one-way ANOVA.

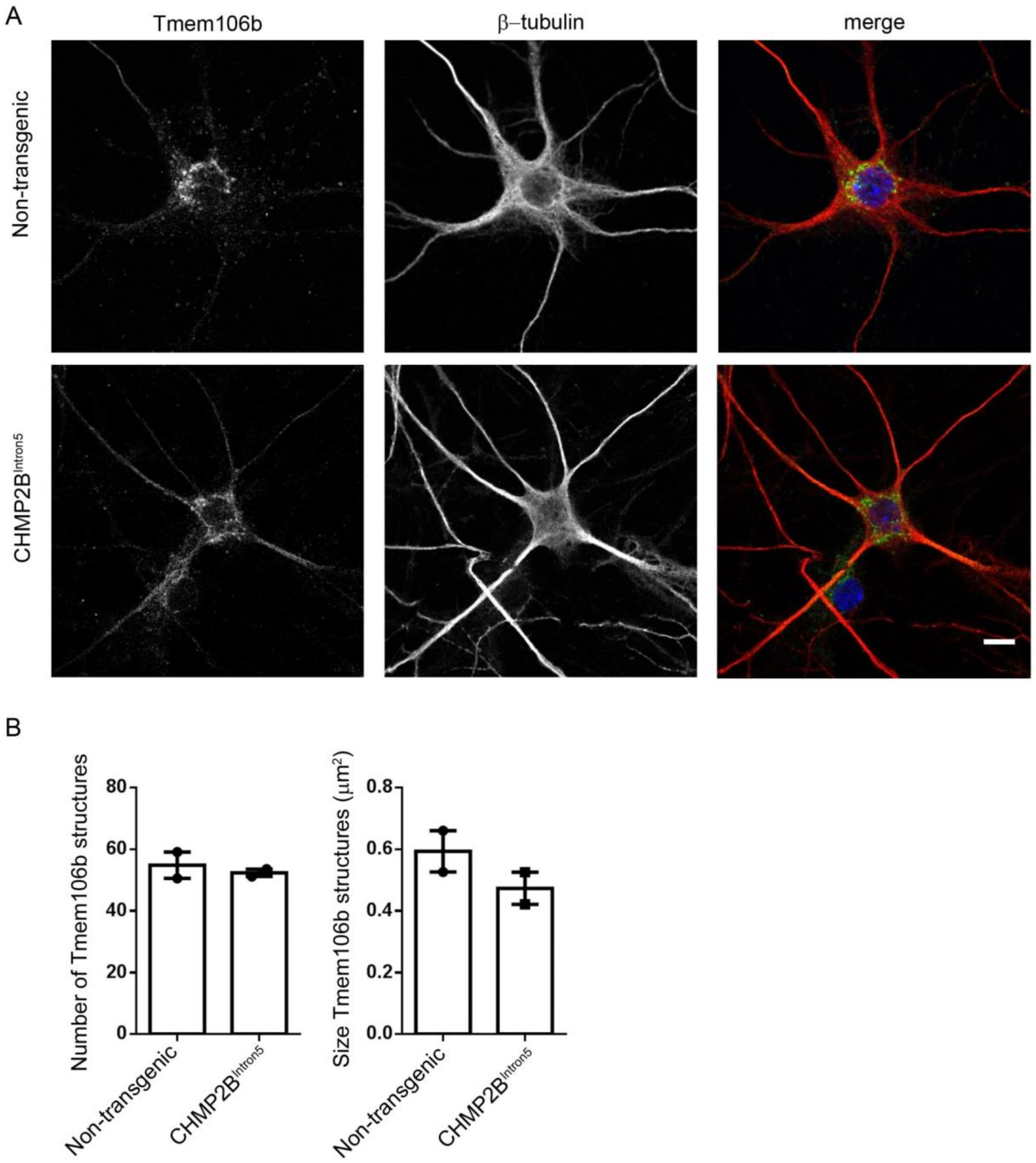

Supplementary Figure 7. Effects of Tmem106b knockdown on LysoTracker trafficking and neurite outgrowth in non-transgenic neurons. (A) Representative images of LysoTracker labelled non-transgenic cortical neurons treated with the indicated ASOs. Scale bar 10 µm. (B) Representative kymographs from the live cell imaging of LysoTracker labelled neurons. Horizontal scale bar = 10 µm, vertical scale bar = 20 seconds (C) Quantification of the proportion of moving LysoTracker labelled structures normalised to control N = 2 with 4 – 5 neurons per N (D) Sholl analysis of ASO treated cortical neurons, representative images found in (E), N = 6 (with 10-12 neurons per N). Scale bar 10 µm.

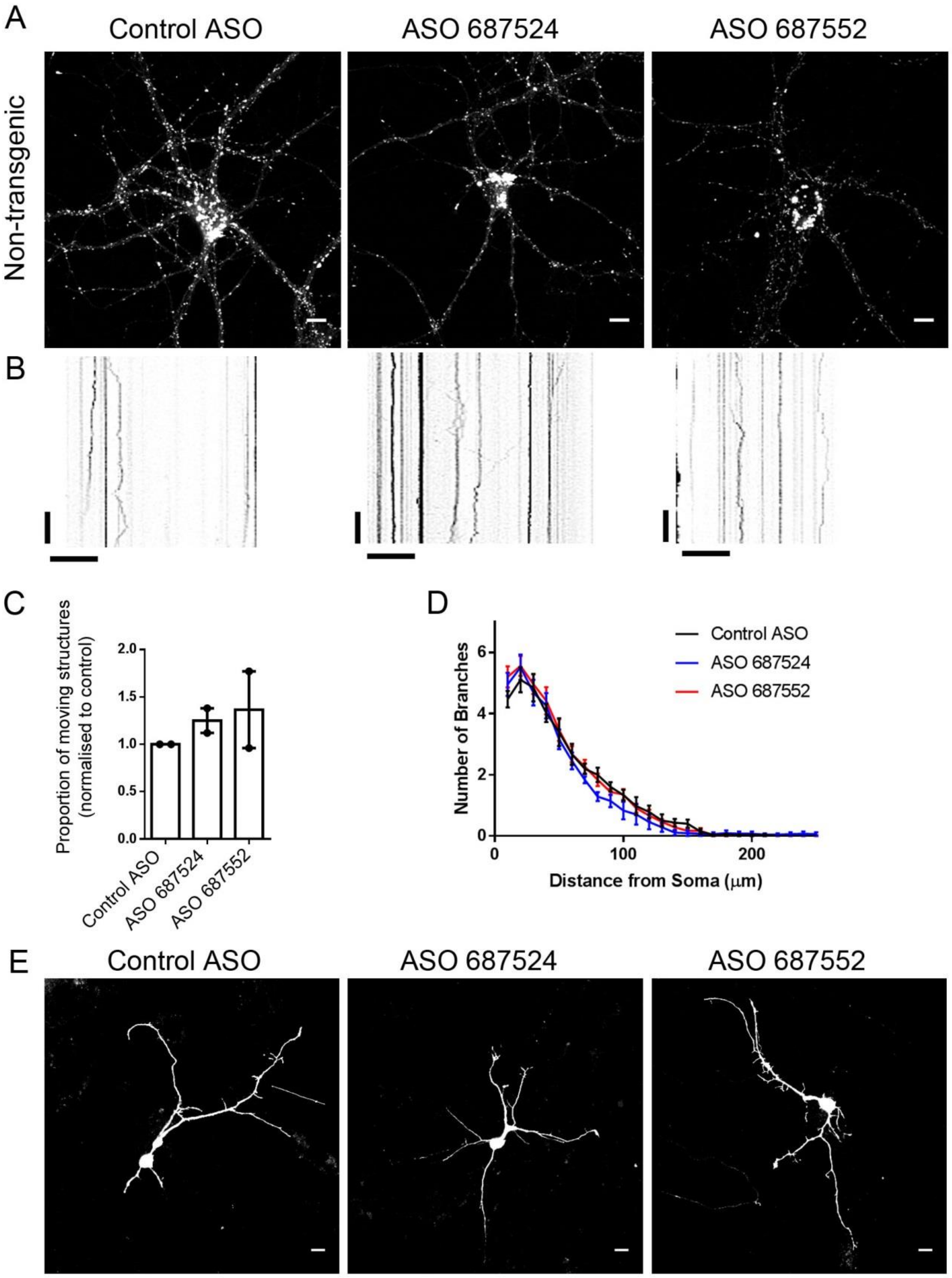

Supplementary Figure 8. Tmem106b localisation in control and mutant CHMP2B neurons. (**A**) Representative images of non-transgenic or mutant CHMP2B cultures stained for Tmem106b and β-tubulin. Scale bar 10 µm. (**B**) Quantification of the size number and size of Tmem106b labelled structures in the soma of non-transgenic and mutant CHMP2B cortical neurons. N = 2 with 5-7 neurons per N.

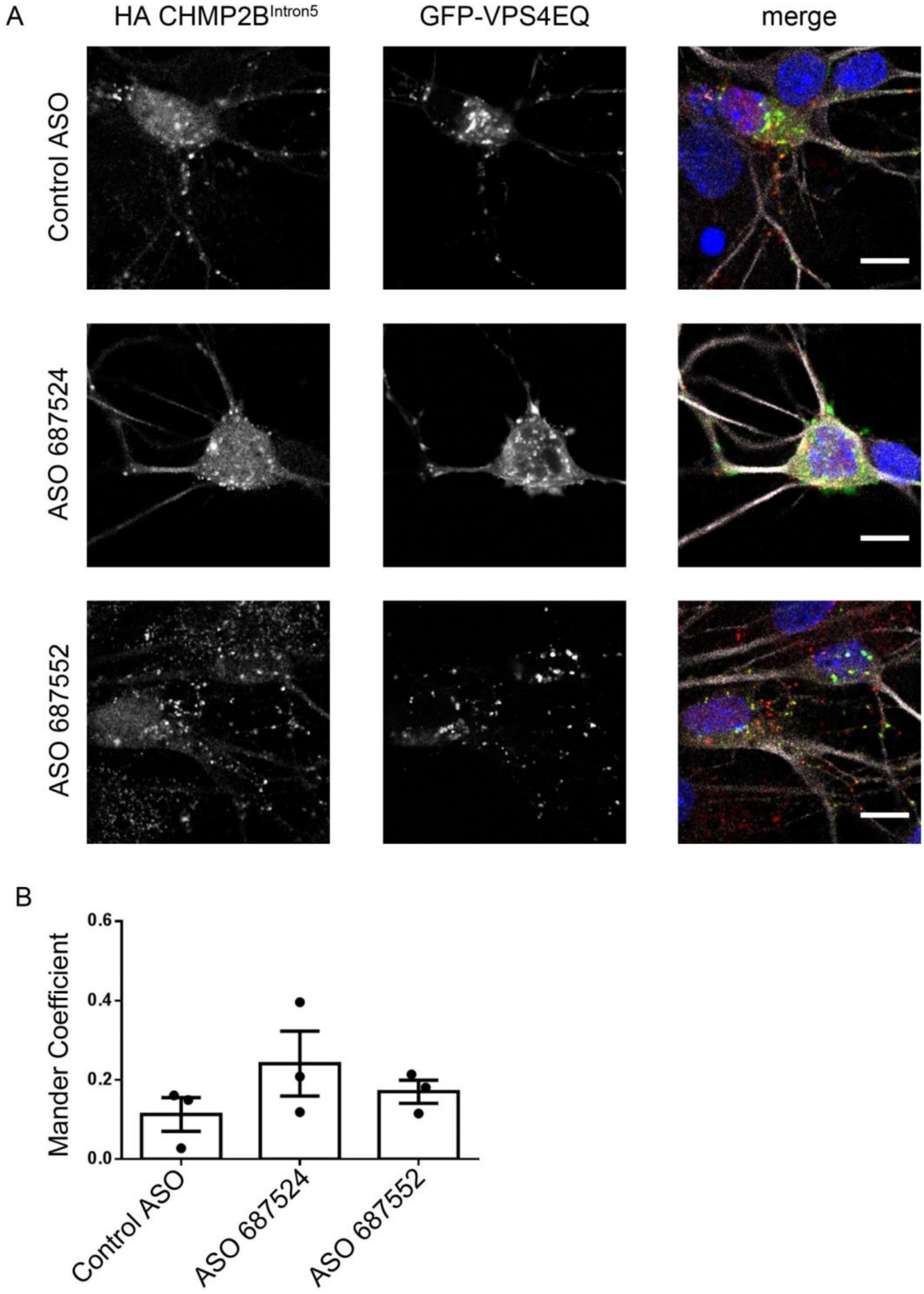

Supplementary Figure 9. Tmem106b knockdown does not affect colocalisation of mutant CHMP2B and VPS4. (**A**) Representative images of primary cortical cultures treated with the indicated Tmem106b targeting or control ASO transfected with HA-tagged mutant CHMP2B and GFP-VPS4EQ. Scale bar = 10 µm. (B) Quantification of colocalisation of HA-mutant CHMP2B and GFP-VPS4EQ. N = 3 DIV 7-10 cultures, with 5 – 10 neurons per N.

Supplementary methods

Autophagy Assay

Cultures were starved by removal of maintenance medium and addition of EBSS. Cultures were then returned to the incubator for 6 hours before processing for staining.

Coverslips were fixed in ice cold methanol for 3 minutes, then blocked in 3 % BSA in PBS for 30 minutes. Primary antibodies were applied in PBS for 1 hour at room temperature followed by 3 washes in PBS. Secondary antibodies were applied in PBS for 1 hour at room temperature, followed by 3 5 minute washes in PBS, and mounted with DAPI. Primary antibodies were LC3 (Nanotools) and WIPI (kind gift form Sharon Tooze).

Fluorescent ASO Internalisation

Primary cortical cultures were incubated with 0.5 or 1 µM of a fluorescein tagged non-targetting ASO (sequence TCCGTCATCGCTCCTCAGGG) at plating. Coverslips were fixed at 3 or 7 days in vitro and immunostained for Tuj-1. Presence or absence of fluorescent ASO in 100 Tuj-1 positive neurons was scored by eye.
